# Causal relationships between gut microbiota and male reproductive inflammation and infertility: Insights from Mendelian randomization

**DOI:** 10.1097/MD.0000000000042323

**Published:** 2025-04-25

**Authors:** Xiaohong Wu, Jingwen Mei, Shicun Qiao, Wen Long, Zhoushan Feng, Guo Feng

**Affiliations:** a Department of Neonatology, Guangzhou Key Laboratory of Neonatal Intestinal Diseases, The Third Affiliated Hospital, Guangzhou Medical University, Guangzhou, China; b Department of Pediatric, The Third Affiliated Hospital, Guangzhou Medical University, Guangzhou, China; c Department of Radiology, The Third Affiliated Hospital, Guangzhou Medical University, Guangzhou, China.

**Keywords:** gut microbiota, infertility, Mendelian randomization, prostatitis, sperm-related proteins

## Abstract

The study observed interactions between gut microbiota and male reproductive health, noting that the causal relationships were previously unclear. It aimed to explore the potential cause-and-effect relationship between gut bacteria and male reproductive problems such as inflammation, infertility, and sperm functionality, using a two-sample Mendelian randomization method to examine these connections. The analysis found that certain bacterial genera, such as Erysipelatoclostridium (0.71 [0.55–0.92]), Parasutterella (0.74 [0.57–0.96]), Ruminococcaceae UCG-009 (0.77 [0.60–0.98]), and Slackia (0.69 [0.49–0.96]), showed protective effects against prostatitis. In contrast, other genera like Faecalibacterium (1.59 [1.08–2.34]), Lachnospiraceae UCG004 (1.64 [1.15–2.34]), Odoribacter (1.68 [1.01–2.81]), Paraprevotella (1.28 [1.03–1.60]), and Sutterella (1.58 [1.13–2.19]) were detrimental. Additionally, causal relationships were identified between 2 genera and orchitis and epididymitis, 3 genera and male infertility, and 5 genera and abnormal spermatozoa. Further analysis of sperm-related proteins revealed causal associations between specific bacterial genera and proteins such as SPACA3, SPACA7, SPAG11A, SPAG11B, SPATA9, SPATA20, and ZPBP4. The results remained robust after sensitivity analysis and reverse Mendelian randomization analysis. The study concluded that specific bacterial genera have causal roles in reproductive inflammation, infertility, and sperm-associated proteins. This provides a novel strategy for the early diagnosis and identification of therapeutic targets in reproductive inflammation and infertility.

## 1. Introduction

Globally, 8% to 12% of couples experience infertility, with male factors contributing to 30% to 50% of cases.^[1]^ A global survey reported that between 1990 and 2017, male infertility rates increased annually by 0.291%.^[2]^ The causes of male infertility are diverse, ranging from congenital issues like chromosomal abnormalities to acquired conditions like prostatitis and orchitis, all of which can affect sperm production.^[3]^ Sperm undergo a series of biochemical reactions involving genetic and protein substances. Poor biochemical combinations are linked to sperm abnormalities such as low count, poor motility, and abnormal morphology.^[4–6]^ These abnormalities highlight the complex interplay of metabolic and genetic factors in male reproductive health.

The human gut hosts trillions of microorganisms, including bacteria, fungi, viruses, and parasites.^[7]^ This ecosystem plays a crucial role in various bodily functions, such as digestion, metabolism, and inflammation, by influencing endocrine, neural, and immune pathways.^[8,9]^ Evidence suggests a close connection between gut microbiota composition and the reproductive system.^[10,11]^ The gut microbiome impacts the male reproductive system through mechanisms like metabolic byproducts, spermatogenesis, and hormonal regulation.^[12]^ It also affects reproductive functions by interacting with the immune and nervous systems, influencing the prostate environment and sperm quality.^[13,14]^ Disruption of gut flora can worsen these immune issues, leading to systemic inflammation and reproductive problems.^[15,16]^ Understanding this relationship across different individuals and conditions is vital for improving male reproductive health. Early prevention, potentially through specific probiotics, offers promising avenues for enhancing health and preventing fertility-related issues.

Previous studies have suggested the gut microbiota as a novel biomarker for predicting and preventing infertility. However, many rely on observational data or case-control designs, which are prone to confounding factors like diet, age, and mental state. This complexity hinders strong causal inferences about the role of gut microbiota in male reproductive health. Mendelian randomization (MR) offers a solution by using genetic variants as instrumental variables (IVs) to assess the causal relationship between exposures (such as changes in gut biota) and reproductive outcomes (such as proteins, inflammation, and sperm abnormalities), reducing the risk of confounding.^[12]^ The random allocation of genotypes at conception strengthens the credibility of causal inferences.^[17]^ MR has been used successfully in studies exploring the causal links between gut microbiota and diseases like immune and endocrine disorders.^[18,19]^ In this study, we used the latest genome-wide association studies data to conduct a two-sample MR analysis. Our goal was to determine if assessing gut microbiome abundance in male reproductive health and infertility can establish new standards and provide more effective preventive or therapeutic interventions for these common male health issues.

## 2. Method

### 2.1. Source of gut microbiome and outcome data

Genetic variations in the gut microbiome were discovered in a large-scale association study involving 24 cohorts with 18,340 participants.^[20]^ Among them, 20 cohorts consisted of samples of single ancestry, with the majority of participants (16 cohorts, N = 13,266) being of European descent. The microbiota quantitative trait locus mapping study for each queue only included taxa present in > 10% of samples, totaling 211 taxa (131 genera, 35 families, 20 orders, 16 classes, and 9 phyla). For better practical application, only genus level data were analyzed, and 12 genera that were not clearly named were excluded, ultimately resulting in the inclusion of 119 classified genera.^[7]^ Primary data regarding male reproductive inflammation (prostatitis, orchitis, and epididymitis) and infertility (abnormal spermatozoa and male infertility) were mainly derived from the FinnGen database. Proteins associated with sperm formation were sourced from the genomic atlas of the human plasma proteome.^[21]^ Additionally, for convenient analysis and data collation and extraction, we acquired the resultant GWAS data directly from the OPEN GWAS website (https://gwas.mrcieu.ac.uk/). Detailed information regarding the collected data, which includes ID numbers, single nucleotide polymorphisms (SNPs), and sample sizes, is provided (Table S1, Supplemental Digital Content, https://links.lww.com/MD/O792).

### 2.2. Study design

**Figure 1.**
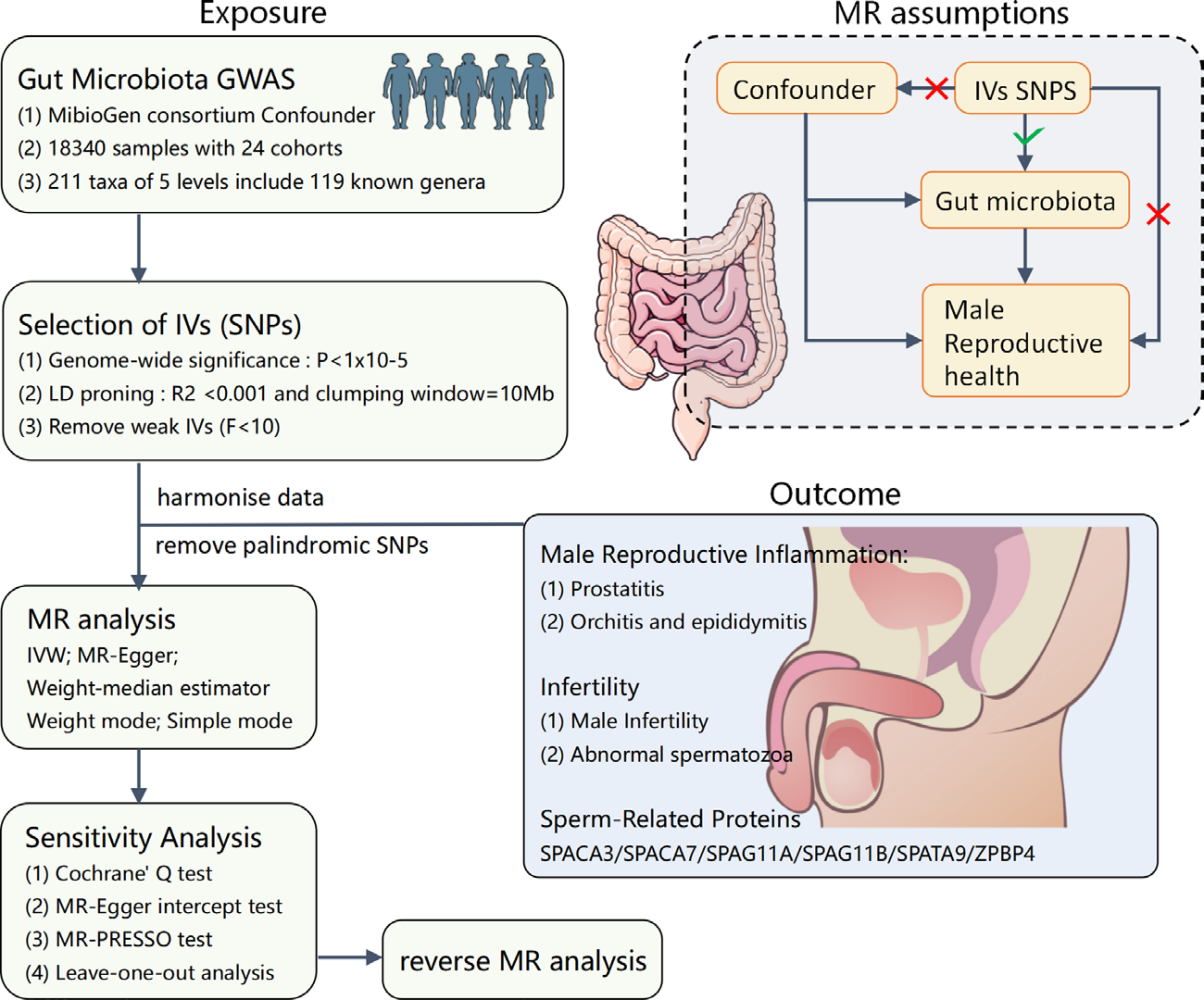
Study design and workflow. GWAS = genome-wide association studies; IVs = instrumental variables; IVW = inverse-variance weighting; MR = Mendelian randomization; SNPs = single nucleotide polymorphisms.

The overall structure of the study is illustrated in Figure 1. We utilized the two-sample MR method to probe the causal association between the entities of the gut microbiome and male reproductive health. To mitigate biases influencing the results maximally, we strived to fulfill 3 crucial assumptions while employing the MR method.^[22]^ First, the relevance assumption stipulates a robust and strong correlation between the IVs and the exposure. Second, the independence assumption posits that IVs should be independent of the confounding factors influencing the “exposure-outcome” relationship. Last, the exclusion restriction assumption asserts that genetic variations can only influence the outcome through the exposure and should not affect the outcome via any alternative pathways.

### 2.3. Ethics statement

Since the data used in this study were ethically approved in their initial research, no additional ethical permission was required for this study.

### 2.4. Criteria for IV selection

Priority is given to SNPs associated with each genus at the genome-wide significance threshold (*P* < 1.0 × 10⁻⁵) as potential strong IVs.^[23]^ To mitigate the bias from weak instruments, the strength of IVs is quantified using the formula F = β²_exposure/SE²_exposure,^[24,25]^ excluding variables with an F-statistic below 10.^[26]^ Furthermore, by setting the R² threshold to 0.001 and maintaining a genetic distance of 10 Mb, variable independence is ensured, and the effect of linkage disequilibrium is reduced. Last, SNPs with minor allele frequencies ≤ 0.01 or those that were palindromic or ambiguous were excluded.

### 2.5. MR analysis

All statistical analyses were conducted using R software version 4.2.3, utilizing the “MR-PRESSO” and “TwoSampleMR” packages.

To investigate causal relationships between exposures and outcomes, we employed a series of methods, including inverse-variance weighting (IVW), MR Egger, weighted median, weighted mode, and simple mode.^[27–29]^ Notably, if inconsistent results were yielded by these methods, the results from the IVW method were considered the primary method. The IVW method is slightly superior under certain conditions compared to other methods.^[29]^ It consolidates Wald ratio estimates from each SNP into a single causal estimate for each hazard ratio, each obtained by dividing the SNP-outcome association by the SNP-exposure association.^[30]^ MR–Egger generally complies with the instrument strength independent of direct effect assumption and accommodates pleiotropy to some extent, reflecting the dose–response relationship between IV and outcomes.^[28,31]^ The weighted median method maximally reduces Type-1 error and allows some invalid genetic variations.^[29]^ Weighted mode and simple mode methods remain reliable when the majority of IVs satisfying causal inference criteria have similar causal estimates. The causal relationships between gut microbiota and diseases are described using odds ratios (ORs) [95% confidence intervals (CIs)]; causal relations with sperm-related proteins are expressed using β [95% CI].^[32,33]^ To enhance the reliability of the results, a series of sensitivity analyses were also performed. Cochran Q test was used to evaluate heterogeneity between IVs, and leave-one-out sensitivity analysis was utilized to assess the impact of outliers on the stability of the results. The presence of horizontal pleiotropy can challenge the second MR assumption; hence, various methods were employed for its detection. Specifically, MR–Egger intercept test and MR-PRESSO global test assessed the presence of horizontal pleiotropy.^[34,35]^ The MR-PRESSO method further adjusted for pleiotropy by identifying and removing outliers, setting permutation counts at 1000 for the analysis.^[36,37]^

To ascertain the existence of bidirectional causal relationships, reverse MR analyses were conducted. In this context, male reproductive tract inflammation, infertility, and sperm-related proteins were treated as exposures, with corresponding SNPs serving as IVs, and identified pathogenic genera were considered outcomes.

## 3. Result

### 3.1. Selection of SNPs

IVs were selected using strict criteria: genome-wide significance (*P* < 1.0 × 10⁻⁵), linkage disequilibrium, validation by F-statistics, and exclusion of palindromic or ambiguous sequences. All IVs had F-statistics well above the threshold of 10, ensuring they were strong and reliable for their associated bacterial taxa (Table S2, Supplemental Digital Content, https://links.lww.com/MD/O792).

### 3.2. Male reproductive inflammation

#### 3.2.1. Prostatitis

**Figure 2.**
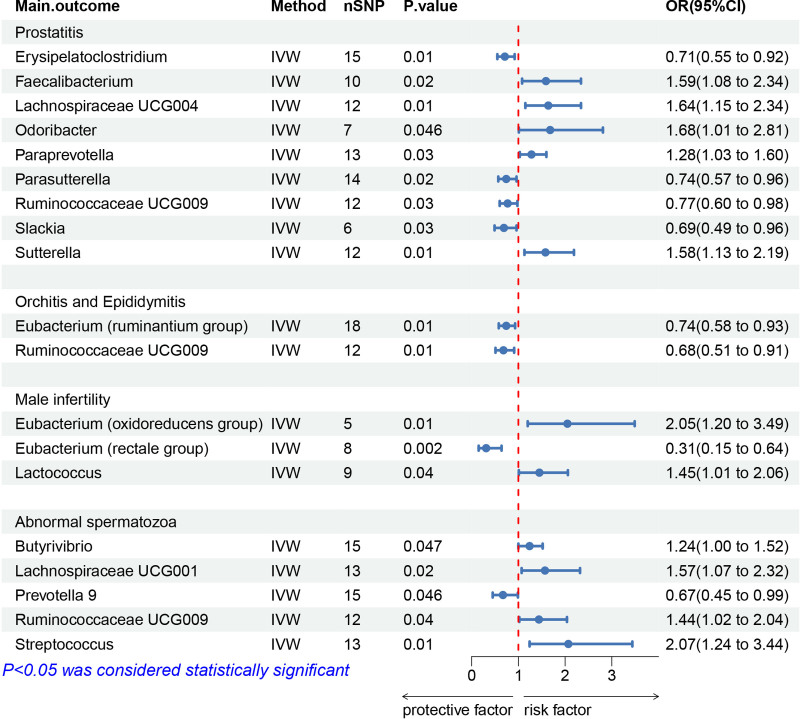
MR results of causal links between gut microbiome and inflammation of the male reproductive inflammation and infertility. CI = confidence intervals; IVW = inverse-variance weighting; OR = odds ratios; SNPs = single nucleotide polymorphisms.

**Figure 3.**
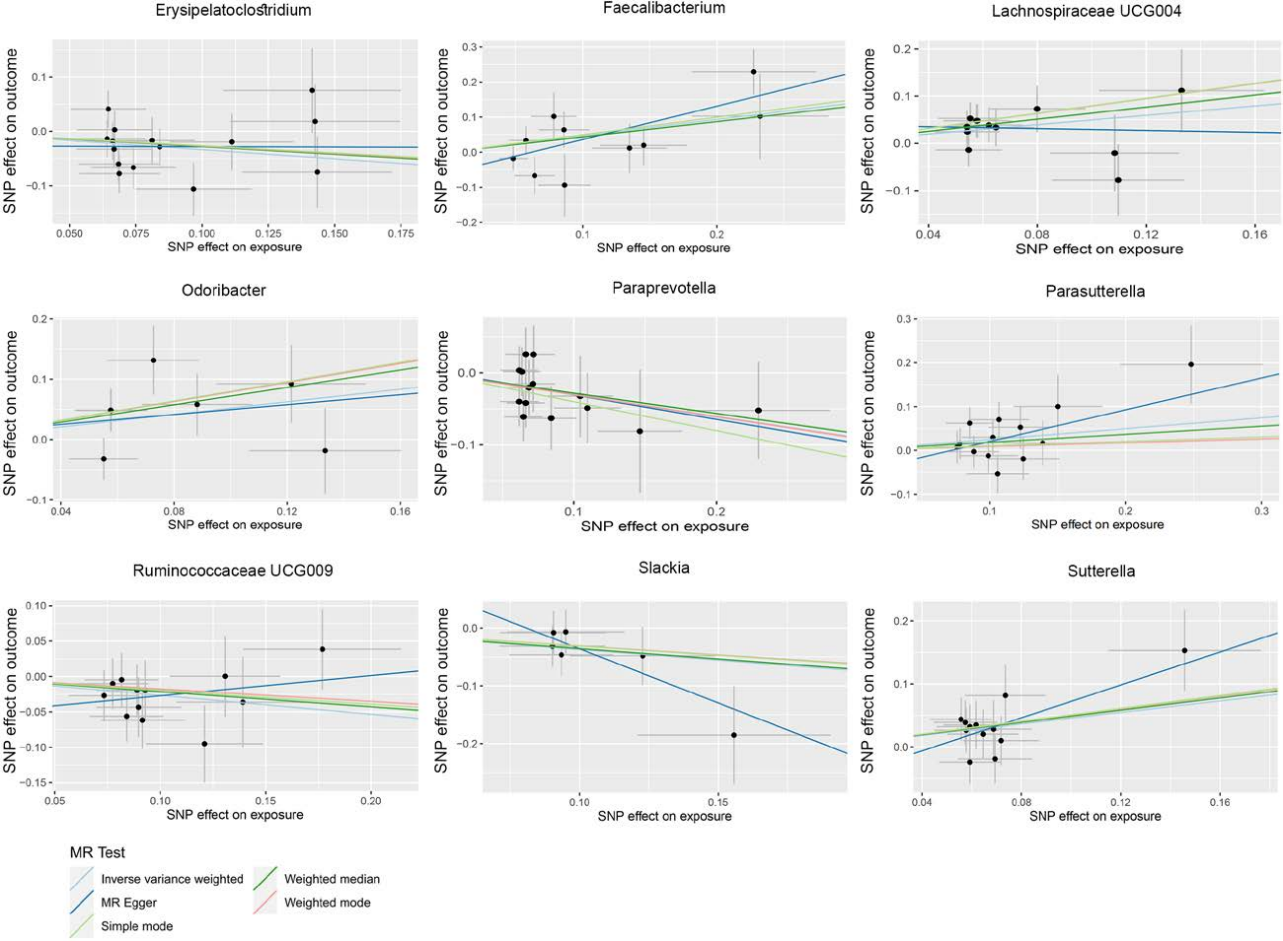
Scatter plot of the causal association between gut microbiome and prostatitis. SNPs = single nucleotide polymorphisms.

Nine bacterial genera have been linked to prostatitis (IVW-*P* < .05). Erysipelatoclostridium, Parasutterella, Ruminococcaceae UCG-009, and Slackia appear to have protective effects with ORs of 0.71 [0.55–0.92], 0.74 [0.57–0.96], 0.77 [0.60–0.98], and 0.69 [0.49–0.96], respectively. In contrast, Faecalibacterium (1.59 [1.08–2.34]), Lachnospiraceae UCG004 (1.64 [1.15–2.34]), Odoribacter (1.68 [1.01–2.81]), Paraprevotella (1.28 [1.03–1.60]), and Sutterella (1.58 [1.13–2.19]) are identified as risk factors for prostatitis (Figs. 2 and 3).

#### 3.2.2. Orchitis and epididymitis

Two bacterial genera, Eubacterium (ruminantium group) and Ruminococcaceae UCG009, have been identified as protective factors against orchitis and epididymitis, showing values of 0.74 [0.58–0.93] and 0.68 [0.51–0.91], respectively (*P* = .01) (Fig. 2; Figure S1, Supplemental Digital Content, https://links.lww.com/MD/O793).

### 3.3. Infertility

#### 3.3.1. Male infertility

Three bacterial genera have been linked to male infertility (IVW-*P* < .05). Eubacterium (rectale group) was associated with a reduced risk (0.31 [0.15–0.64]), while Eubacterium (oxidoreducens group) (1.58 [1.13–2.19]) and Lactococcus (1.45 [1.01–2.06]) were associated with an increased risk (Fig. 2; Figure S2, Supplemental Digital Content, https://links.lww.com/MD/O793).

#### 3.3.2. Abnormal spermatozoa

IVW analysis identified 5 bacterial genera significantly associated with abnormal spermatozoa (IVW-*P* < .05). Butyrivibrio (1.24 [1.00–1.52]), Lachnospiraceae UCG001 (1.57 [1.07–2.32]), Ruminococcaceae UCG009 (1.44 [1.02–2.04]), and Streptococcus (2.07 [1.24–3.44]) were linked to an increased risk, while Prevotella 9 (0.67 [0.45–0.99]) was associated with a reduced risk (Fig. 2; Figure S3, Supplemental Digital Content, https://links.lww.com/MD/O793).

### 3.4. Sperm-related proteins

The study found several key associations between bacterial genera and sperm-related proteins: Defluviitaleaceae UCG011 was positively linked to SPACA3 (β = 0.23), while Rikenellaceae RC9 was negatively correlated. Peptococcus had a positive association with SPACA7 (β = 0.17), whereas Anaerostipes was negatively associated (β = −0.15). Oxalobacter was positively linked to SPAG11A (β = 0.16), while Anaerostipes showed a negative association (β = −0.27). Candidatus_Soleaferrea and Coprococcus 2 were negatively associated with SPAG11B (β = −0.29 and −0.31), while Anaerotruncus, Blautia, and Eubacterium (brachy group) had positive associations (β = 0.41, 0.28, and 0.17, respectively).

**Figure 4.**
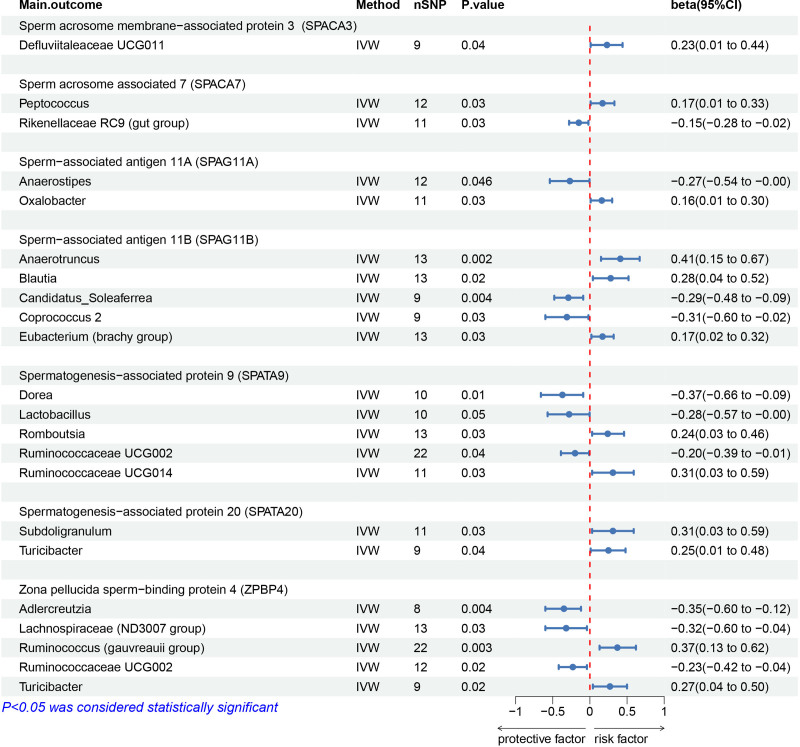
MR results of causal links between gut microbiome and sperm-related proteins. CI = confidence intervals; IVW = inverse-variance weighting; OR = odds ratios; SNPs = single nucleotide polymorphisms.

Dorea, Lactobacillus, and Ruminococcaceae UCG002 were negatively associated with SPATA9 (β = −0.37, −0.28, and −0.20), while Romboutsia and Ruminococcaceae UCG014 were positively associated (β = 0.24 and 0.31). Subdoligranulum and Turicibacter had positive associations with SPATA20 (β = 0.31 and 0.25). Adlercreutzia, Lachnoclostridium, and Ruminococcaceae UCG002 showed negative associations with ZPBP4 (β = −0.35, −0.32, and −0.23), while Lachnospiraceae (ND3007 group), Ruminococcus (gauvreauii group), and Turicibacter were positively associated with ZPBP4 (β = 0.61, 0.37, and 0.27) (Fig. 4; Figures S4–S10, Supplemental Digital Content, https://links.lww.com/MD/O793).

### 3.5. Sensitivity analysis

**Figure 5.**
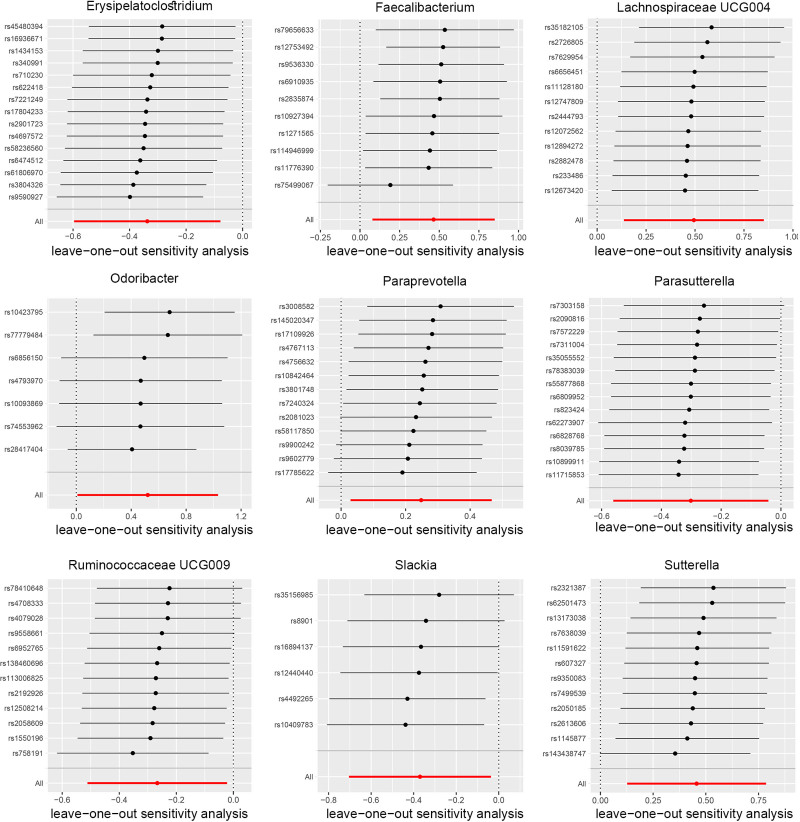
Leave-one-out analysis of the causal association between gut microbiome and prostatitis.

The other MR methods, such as MR Egger, weighted median, weighted mode, and simple mode, might not all have *P* values below .05, but the overall trend is consistent with the IVW results (Tables S3–S13, Supplemental Digital Content, https://links.lww.com/MD/O792) (Fig. 3; Figures S1–S10, Supplemental Digital Content, https://links.lww.com/MD/O793). No heterogeneity was detected by Cochran Q test (Table S14, Supplemental Digital Content, https://links.lww.com/MD/O792). Employing the MR–Egger intercept and MR-PRESSO analysis revealed no signs of pleiotropy (Tables S15–S16, Supplemental Digital Content, https://links.lww.com/MD/O792). Following the exclusion of 1 SNP per case, no notable alterations were observed in the estimated relationship between gut microbiota and inflammation, infertility and sperm-related proteins (Fig. 5; Figures S11–S20, Supplemental Digital Content, https://links.lww.com/MD/O793).

### 3.6. Bidirectional MR analysis

When positive results were obtained from the initial MR analysis, a subsequent bidirectional MR analysis was performed. A causal link was observed between male infertility and Lactococcus (OR = 1.05, 95% CI = 1.00–1.11, *P* = .048) and between SPAG11B and Coprococcus 2 (β = 0.08, 95% CI = 0.01–0.14, *P* = .02) but not with other gut microbiota genera. Sensitivity analyses revealed no heterogeneity or pleiotropy (data not shown).

## 4. Discussion

In the MR analysis of gut microbiota and male reproductive health, we explored the causal relationships between gut microbiota traits and conditions like male infertility, reproductive inflammation, and sperm-associated proteins. We identified 9 bacterial genera linked to prostatitis, 2 to orchitis and epididymitis, 3 to male infertility, and 5 to abnormal sperm. Additionally, different bacteria were associated with sperm-related proteins. Our study overcomes weak IV bias and ensures robust results by using various MR methods, Cochran Q tests, and MR-PRESSO analysis. Unlike previous studies, which mainly focused on correlations, our research systematically investigates the causal relationships between gut microbiota and male reproductive health.

The composition of microbial communities is intricately linked to prostate health, encompassing conditions such as prostatitis and prostate cancer. These communities include gut microbiota, urinary tract microbiota, and oral microbiota.^[13]^ Our MR analysis identified certain bacteria with a causal association with prostatitis, reinforcing the link between gut microbiota and prostate health. The gut microbiota modulates Th17/Treg balance, influencing prostatitis, and alterations in gut microbiota – whether through dietary changes or imbalances – can impact prostate health.^[38–40]^ Specifically, Faecalibacterium was identified as a detrimental factor for the prostate. Despite being one of the most prevalent gut bacteria in healthy adults and considered beneficial for inflammatory bowel disease and kidney disease, its role in prostate health appears to be adverse.^[41–43]^ Conversely, Parasutterella was found to have a protective effect against prostatitis and other inflammatory conditions, such as ulcerative colitis. This suggests that certain gut bacteria exert broad anti-inflammatory effects, underscoring the complex and context-dependent roles of gut microbiota in various health conditions.^[44,45]^

This study uncovers a causal connection between gut microbiota and the testicular axis. We found that Ruminococcaceae UCG009 and Eubacterium (ruminantium group) exert protective effects, with Ruminococcaceae UCG009 also providing protection against prostatitis and respiratory infections, underscoring its strong anti-inflammatory properties.^[46]^ The gut microbiota is recognized as an endocrine organ influencing distant organs, with metabolic factors linked to infertility through the gut-brain and gut-testicular axes.^[47,48]^ For example, decreased bile acid levels impair vitamin A absorption, reducing Ruminococcaceae abundance and leading to abnormal sperm development.^[49]^ Additionally, the gut microbiota impacts testicular inflammation by modulating immune cells, cytokines, and chemokines. Given the rising antibiotic resistance and treatment challenges for orchitis and epididymitis due to the blood–testis barrier, early identification and modulation of gut microbiota offer significant potential for preventive and therapeutic applications.^[50,51]^

In addition to its link with inflammatory factors, the gut microbiota can have a direct causal relationship with male infertility. Surprisingly, Lactococcus has been identified as a risk factor for male infertility, despite its role as a protective factor against female infertility and its presence in female reproductive health microbiota.^[52–54]^ This discrepancy may be related to endocrine factors, such as sex hormones. Research shows that sex differences impact gut microbiota composition, influencing conditions like systemic lupus erythematosus and type-1 diabetes.^[55–57]^ Androgens can significantly alter the gut microbiota, while the microbiota also affects androgen production and metabolism. For example, transplanting microbiota from adult male mice to immature female mice has been shown to increase testosterone levels and induce metabolic changes.^[12,58,59]^

The most direct link to male infertility is often related to sperm abnormalities, such as oligospermia, asthenospermia, and azoospermia. In this study, Prevotella 9 was identified as a protective genus against male sperm abnormalities, potentially due to its positive correlation with higher testosterone levels.^[60]^ Conversely, semen containing micrococci or α-hemolytic streptococci is associated with increased rates of oligospermia and teratospermia, as well as lower sperm concentration and percentage of normal sperm compared to uninfected semen.^[61]^ Additionally, significant differences in β-diversity were observed in the microbiota of patients with asthenospermia and oligospermia, with higher relative abundances of Spermatococcus, Lactobacillus, and Roseburia in asthenospermic patients, and notably higher Lactobacillus levels in oligospermic patients.^[62]^

This study reveals that the gut microbiota can influence proteins critical to sperm structure and function, including SPACA3, SPACA7, SPAG11A, SPAG11B, SPATA9, SPATA20, and ZPBP4, all of which are involved in sperm formation, maturation, and the sperm-egg recognition process. Through MR analysis, we identified varying effects of different gut microbiota on these proteins. While the underlying mechanisms require further investigation, our findings establish a causal link between gut microbiota and sperm-associated proteins, providing potential strategies for improving sperm quality through targeted interventions.

While much remains to be understood about the specific roles of gut microbiota in male reproductive health, their impact can be viewed from several key perspectives. The gut microbiota’s ability to modulate immune responses may influence sperm quality.^[63]^ Additionally, bioactive molecules and metabolic products produced by these bacteria could affect hormone balance and reproductive organ function in males.^[64]^ Gut bacteria are integral to the metabolism of male hormones and can contribute to conditions like prostatitis and orchitis through systemic inflammation.^[12,65]^ Moreover, gut microbiota is linked to mental health, where stress and mood disorders can alter hormone levels and impact fertility.^[66,67]^ Finally, gut bacteria play a crucial role in nutrient absorption, including trace elements like zinc and selenium, which are vital for sperm production and maturation.^[68]^

## 5. Advantages and limitations

Integrating genetic information is crucial for advancing disease prevention and treatment, but translating this knowledge into clinical practice remains challenging. This study established a connection between gut microbiota and male reproductive health using MR as a tool to treat genetic information as IVs. MR analysis effectively mitigated confounding factors and reverse causality, providing a cost-efficient alternative to randomized controlled trials.^[69,70]^ Various statistical methods, including MR-PRESSO and MR-Egger regression intercept tests, ensured result consistency and addressed horizontal pleiotropy, while inverse MR clarified potential reverse causal relationships. By employing a two-sample MR design with nonoverlapping data, the study minimized bias and provided a robust framework for exploring the complex relationship between gut microbiota and reproductive health.^[71]^ The findings underscore the importance of gut microbiota in male infertility and suggest potential preventive and therapeutic strategies through dietary changes, microbial supplements, or fecal microbiota transplantation.^[72]^

When examining the relationship between gut microbiota and male infertility, certain limitations must be considered, as they could affect our interpretation of the findings. Our research primarily utilizes GWAS summary data from individuals of European ancestry, leading to potential biases and limiting the generalizability of the results across other ethnicities. Although sex factors were adjusted and genetic variants on sex chromosomes excluded, sex-specific biases cannot be entirely ruled out. The bacterial classification at the genus level may reduce the precision of the analysis, and the study did not explore the link between male reproductive system inflammation and infertility in depth. Additionally, SNPs not meeting the conventional GWAS significance threshold (*P* < 5 × 10^−8^) might have introduced bias.^[73]^ To gain a more comprehensive understanding of the causal relationship between gut microbiota and male infertility, future research should address these limitations by including more diverse populations, employing finer bacterial classifications, and leveraging multiomics approaches to explore the complex gene-environment interactions that influence male reproductive health.

## 6. Conclusion

This study used genetic data from gut microbiota and MR analysis to explore causal links with male reproductive health, including sperm-related proteins, inflammation, and infertility. Specific bacterial genera were identified as having causal effects on risks, and the robustness of these findings has been rigorously tested. By analyzing at the genetic level, this research reveals the relationship between gut microbiota and male reproductive health, offering a novel strategy for the early diagnosis of patients and identification of therapeutic targets in reproductive inflammation and infertility.

## Acknowledgments

We extend our sincere gratitude to the researchers who provided the GWAS summary statistical data used in this study. We also appreciate the assistance of American Journal Experts for their editorial support.

## Author contributions

**Conceptualization:** Zhoushan Feng, Guo Feng.

**Data curation:** Jingwen Mei, Shicun Qiao, Wen Long.

**Formal analysis:** Shicun Qiao, Wen Long.

**Investigation:** Jingwen Mei, Shicun Qiao.

**Methodology:** Jingwen Mei, Shicun Qiao, Guo Feng.

**Project administration:** Xiaohong Wu, Zhoushan Feng.

**Resources:** Xiaohong Wu, Shicun Qiao, Zhoushan Feng, Guo Feng.

**Software:** Jingwen Mei.

**Supervision:** Xiaohong Wu, Zhoushan Feng.

**Validation:** Wen Long, Guo Feng.

**Visualization:** Wen Long.

**Writing – original draft:** Jingwen Mei, Wen Long.

**Writing – review & editing:** Xiaohong Wu, Zhoushan Feng, Guo Feng.

## Supplementary Material


