# Supplementary material for: Causal relationships between gut microbiota and male reproductive inflammation and infertility: Insights from Mendelian randomization
Source: Medicine (Baltimore). 2025 Apr 25;104(17):e42323. doi: 10.1097/MD.0000000000042323 (PMC12039986; doi:10.1097/MD.0000000000042323)
Supplement: Supplementary file 1 [file medi-104-e42323-s001.pdf]

Table S1, Supplemental Digital Content. Data source and GWAS sample size for male reproductive health

Disease	ID number (https://gwas.mrcieu.ac.uk/)	Simple size (n)	Number of SNPs (n)
**Inflammation**			
Prostatitis	finn-b-N14_PROSTATITIS	74,658	16,377,460
Orchitis and epididymitis	finn-b-N14_ORHCEPINDIDYMITIS	74,096	16,377,371
**Infertility**			
Male infertility	finn-b-N14_MALEINFERT	73,479	16,377,329
Abnormal spermatozoa	finn-b-R18_ABNORMAL_SPERMATOZ	209,921	16,380,442
**Sperm related proteins**			
Sperm acrosome membrane-associated protein 3 (SPACA3)	prot-a-2804	3,301	10,534,735
Sperm acrosome associated 7 (SPACA7)	prot-a-2805	3,301	10,534,735
Sperm-associated antigen 11A (SPAG11A)	prot-a-2806	3,301	10,534,735
Sperm-associated antigen 11B (SPAG11B)	prot-a-2807	3,301	10,534,735
Spermatogenesis-associated protein 9 (SPATA9)	prot-a-2814	3,301	10,534,735
Spermatogenesis-associated protein 20 (SPATA20)	prot-a-2813	3,301	10,534,735
Zona pellucida sperm-binding protein 4 (ZPBP4)	prot-a-3281	3,301	10,534,735

Table S2, Supplemental Digital Content. Utilizing instrumental variables in MR analysis to explore the link between gut microbiota and male reproductive health

Outcome	Bacterial (exposure)	SNP	Effect allele	Other allele	F	Exposure			Outcome		
						Beta	SE	P-value	Beta	SE	P-value
**Inflammation**											
**Prostatitis**											
1	**Erysipelatoclostridium**	rs1434153	G	A	20.163	-0.068	0.015	7.11E-06	0.060	0.034	0.080
2		rs16936671	C	T	19.739	-0.097	0.022	8.88E-06	0.106	0.049	0.031
3		rs17804233	T	C	21.133	-0.066	0.014	4.28E-06	0.018	0.034	0.590
4		rs2901723	C	A	19.755	0.064	0.014	8.80E-06	-0.014	0.034	0.687
5		rs340991	A	G	21.718	-0.074	0.016	3.16E-06	0.067	0.038	0.081
6		rs3804326	A	G	17.691	0.141	0.034	2.60E-05	0.075	0.078	0.337
7		rs45480394	T	G	20.480	-0.069	0.015	6.02E-06	0.078	0.035	0.028
8		rs4697572	A	G	24.640	-0.081	0.016	6.91E-07	0.017	0.043	0.693
9		rs58236560	G	T	22.483	-0.111	0.023	2.12E-06	0.020	0.052	0.706
10		rs61806970	C	T	19.759	0.143	0.032	8.79E-06	0.018	0.067	0.789
11		rs622418	A	G	21.756	-0.067	0.014	3.10E-06	0.033	0.034	0.333
12		rs6474512	A	C	21.900	0.067	0.014	2.87E-06	0.003	0.035	0.933
13		rs710230	T	C	25.941	0.143	0.028	3.52E-07	-0.075	0.065	0.250
14		rs7221249	A	G	34.619	0.084	0.014	4.01E-09	-0.029	0.034	0.399
15		rs9590927	G	A	20.272	-0.065	0.014	6.72E-06	-0.041	0.034	0.228
1	**Faecalibacterium**	rs10927394	G	T	20.549	-0.232	0.051	5.81E-06	-0.103	0.122	0.399
2		rs114946999	C	T	20.649	-0.086	0.019	5.52E-06	-0.064	0.051	0.212
3		rs11776390	T	C	20.793	-0.078	0.017	5.12E-06	-0.102	0.068	0.135
4		rs1271565	C	T	23.195	-0.058	0.012	1.46E-06	-0.034	0.039	0.382
5		rs12753492	A	C	18.295	0.064	0.015	1.89E-05	-0.067	0.054	0.215
6		rs2835874	T	C	19.440	-0.087	0.020	1.04E-05	0.094	0.090	0.299
7		rs6910935	A	G	23.699	0.135	0.028	1.13E-06	0.012	0.071	0.870
8		rs75499067	C	T	23.900	0.228	0.047	1.01E-06	0.229	0.065	0.000
9		rs79656633	T	C	20.326	0.146	0.032	6.53E-06	0.020	0.057	0.730
10		rs9536330	T	C	20.026	-0.048	0.011	7.64E-06	0.018	0.034	0.600
1	**Lachnospiraceae**	rs11128180	A	G	21.404	0.065	0.014	3.72E-06	0.034	0.040	0.401

2	**UCG004**	rs12072562	T	C	19.225	0.133	0.030	1.16E-05	0.112	0.088	0.205
3		rs12673420	G	A	21.930	0.055	0.012	2.83E-06	0.053	0.034	0.119
4		rs12747809	G	A	24.489	-0.062	0.013	7.47E-07	-0.039	0.037	0.303
5		rs12894272	A	G	21.436	0.058	0.013	3.66E-06	0.048	0.036	0.180
6		rs233486	A	G	20.231	-0.080	0.018	6.86E-06	-0.073	0.049	0.134
7		rs2444793	C	T	21.064	-0.054	0.012	4.44E-06	-0.035	0.035	0.308
8		rs2726805	A	G	20.594	0.055	0.012	5.68E-06	-0.014	0.034	0.684
9		rs2882478	G	A	23.781	-0.058	0.012	1.08E-06	-0.048	0.034	0.161
10		rs35182105	A	G	20.522	-0.110	0.024	5.89E-06	0.078	0.076	0.304
11		rs6656451	C	T	20.703	-0.054	0.012	5.36E-06	-0.025	0.034	0.469
12		rs7629954	A	G	20.717	0.108	0.024	5.32E-06	-0.021	0.081	0.800
1		rs10093869	A	G	21.234	-0.058	0.013	4.07E-06	-0.048	0.035	0.164
2		rs10423795	C	T	20.657	0.055	0.012	5.49E-06	-0.032	0.035	0.361
3		rs28417404	A	G	20.290	-0.073	0.016	6.65E-06	-0.131	0.057	0.022
4	**Odoribacter**	rs4793970	A	G	19.912	-0.058	0.013	8.11E-06	-0.049	0.035	0.168
5		rs6856150	G	A	20.635	0.088	0.019	5.56E-06	0.058	0.051	0.257
6		rs74553962	T	G	21.146	0.121	0.026	4.26E-06	0.092	0.064	0.151
7		rs77779484	G	A	24.713	-0.133	0.027	6.65E-07	0.019	0.071	0.793
1		rs10842464	T	C	19.305	-0.076	0.017	1.11E-05	-0.008	0.037	0.838
2		rs145020347	A	G	22.579	-0.125	0.026	2.02E-06	0.019	0.048	0.690
3		rs17109926	A	G	20.903	-0.099	0.022	4.83E-06	0.012	0.038	0.745
4		rs17785622	A	G	22.385	0.248	0.052	2.23E-06	0.196	0.089	0.028
5		rs2081023	A	G	26.854	-0.123	0.024	2.19E-07	-0.053	0.048	0.275
6		rs3008582	T	C	21.643	0.106	0.023	3.28E-06	-0.054	0.044	0.215
7	**Paraprevotella**	rs3801748	G	A	20.624	0.078	0.017	5.59E-06	0.015	0.035	0.666
8		rs4756632	G	T	22.959	-0.139	0.029	1.65E-06	-0.017	0.051	0.743
9		rs4767113	C	T	23.047	0.088	0.018	1.58E-06	-0.003	0.036	0.933
10		rs58117850	C	A	21.427	-0.150	0.032	3.68E-06	-0.100	0.072	0.165
11		rs7240324	T	G	20.315	-0.102	0.023	6.57E-06	-0.030	0.039	0.450
12		rs9602779	A	C	23.463	-0.107	0.022	1.27E-06	-0.070	0.040	0.075
13		rs9900242	A	G	23.699	-0.085	0.018	1.13E-06	-0.062	0.036	0.082

1		rs10899911	A	G	23.429	-0.072	0.015	1.30E-06	-0.026	0.040	0.521
2		rs11715853	G	A	20.586	-0.066	0.015	5.70E-06	-0.026	0.037	0.485
3		rs2090816	A	C	22.494	0.084	0.018	2.11E-06	-0.063	0.044	0.155
4		rs35055552	T	C	21.653	0.110	0.024	3.27E-06	-0.049	0.049	0.319
5		rs55877868	A	C	20.974	-0.104	0.023	4.66E-06	0.032	0.057	0.566
6		rs62273907	A	G	20.873	0.229	0.050	4.91E-06	-0.052	0.067	0.439
7	**Parasutterella**	rs6809952	G	A	20.606	-0.068	0.015	5.64E-06	0.021	0.039	0.595
8		rs6828768	C	T	23.051	0.064	0.013	1.58E-06	0.002	0.034	0.964
9		rs7303158	C	T	23.214	0.065	0.013	1.45E-06	-0.061	0.034	0.072
10		rs7311004	T	C	20.485	-0.062	0.014	6.01E-06	0.040	0.034	0.243
11		rs7572229	G	A	24.929	0.066	0.013	5.95E-07	-0.042	0.034	0.218
12		rs78383039	T	C	24.250	-0.146	0.030	8.46E-07	0.081	0.086	0.343
13		rs8039785	T	G	21.615	0.062	0.013	3.33E-06	0.003	0.034	0.920
14		rs823424	G	A	20.661	-0.071	0.016	5.48E-06	0.015	0.039	0.694
1		rs113006825	T	C	19.937	-0.093	0.021	8.00E-06	0.019	0.042	0.641
2		rs12508214	C	T	21.026	-0.077	0.017	4.53E-06	0.010	0.036	0.780
3		rs138460696	A	G	19.451	0.139	0.032	1.03E-05	-0.036	0.064	0.570
4		rs1550196	G	A	24.849	0.131	0.026	6.20E-07	0.000	0.057	0.996
5		rs2058609	A	G	21.837	0.082	0.017	2.97E-06	-0.005	0.038	0.900
6	**Ruminococcaceae**	rs2192926	A	G	21.290	-0.089	0.019	3.95E-06	0.019	0.036	0.594
7	**UCG009**	rs4079028	C	T	21.094	0.092	0.020	4.37E-06	-0.062	0.040	0.120
8		rs4708333	T	G	23.122	-0.084	0.017	1.52E-06	0.056	0.036	0.118
9		rs6952765	G	A	19.269	0.073	0.017	1.14E-05	-0.027	0.037	0.457
10		rs758191	T	G	22.270	0.177	0.038	2.37E-06	0.039	0.057	0.500
11		rs78410648	A	G	19.034	0.121	0.028	1.28E-05	-0.095	0.055	0.082
12		rs9558661	T	C	20.004	-0.090	0.020	7.73E-06	0.044	0.042	0.305
1		rs10409783	A	G	20.261	0.095	0.021	6.76E-06	-0.007	0.038	0.860
2		rs12440440	A	G	22.397	0.090	0.019	2.22E-06	-0.032	0.036	0.378
3	**Slackia**	rs16894137	C	T	21.791	-0.123	0.026	3.04E-06	0.048	0.050	0.336
4		rs35156985	T	C	20.010	-0.156	0.035	7.70E-06	0.185	0.084	0.028
5		rs4492265	A	G	22.334	-0.091	0.019	2.29E-06	0.008	0.037	0.829

6		rs8901	C	T	25.028	0.093	0.019	5.65E-07	-0.046	0.037	0.212
1		rs1145877	A	G	20.507	-0.074	0.016	5.94E-06	-0.082	0.049	0.097
2		rs11591622	T	G	20.680	-0.069	0.015	5.43E-06	-0.028	0.046	0.541
3		rs13173038	A	G	22.428	-0.072	0.015	2.18E-06	-0.010	0.039	0.797
4		rs143438747	T	C	22.572	-0.146	0.031	2.02E-06	-0.153	0.064	0.017
5		rs2050185	G	A	19.950	0.058	0.013	7.95E-06	0.039	0.035	0.267
6	**Sutterella**	rs2321387	G	A	22.674	-0.059	0.012	1.92E-06	0.024	0.034	0.480
7		rs2613606	C	T	20.124	-0.056	0.012	7.26E-06	-0.044	0.034	0.203
8		rs607327	C	T	20.083	0.058	0.013	7.42E-06	0.026	0.035	0.450
9		rs62501473	G	A	21.588	0.069	0.015	3.38E-06	-0.019	0.039	0.619
10		rs7499539	A	G	22.218	0.062	0.013	2.43E-06	0.035	0.039	0.366
11		rs7638039	T	C	20.133	0.065	0.014	7.22E-06	0.020	0.039	0.605
12		rs9350083	T	G	19.609	-0.059	0.013	9.50E-06	-0.032	0.035	0.366
**Orchitis and epididymitis**											
1		rs10131724	A	C	23.234	-0.200	0.041	1.43E-06	0.011	0.068	0.871
2		rs10923018	G	A	20.378	0.073	0.016	6.36E-06	-0.053	0.041	0.191
3		rs11637981	G	T	20.733	-0.073	0.016	5.28E-06	0.023	0.040	0.561
4		rs13025464	T	C	20.252	-0.074	0.016	6.79E-06	-0.047	0.041	0.248
5		rs139749	C	T	24.219	-0.085	0.017	8.60E-07	-0.001	0.042	0.991
6		rs16891896	G	A	20.027	-0.175	0.039	7.64E-06	0.005	0.072	0.946
7		rs17519472	C	T	21.228	0.108	0.023	4.08E-06	0.001	0.058	0.983
8	**Eubacterium (ruminantium group)**	rs209813	G	A	19.166	-0.103	0.024	1.20E-05	0.093	0.057	0.103
9		rs2116427	A	G	24.983	0.091	0.018	5.78E-07	-0.017	0.046	0.707
10		rs2229917	A	G	22.467	0.154	0.032	2.14E-06	-0.114	0.100	0.253
11		rs2418654	C	T	20.388	-0.075	0.017	6.32E-06	0.038	0.041	0.351
12		rs2817174	C	T	20.125	-0.073	0.016	7.26E-06	0.043	0.041	0.294
13		rs57340348	T	C	21.311	-0.098	0.021	3.91E-06	0.040	0.050	0.432
14		rs606117	A	G	21.296	0.083	0.018	3.94E-06	-0.065	0.045	0.150
15		rs6676699	G	T	20.439	-0.089	0.020	6.16E-06	0.027	0.044	0.535
16		rs7000472	A	G	21.284	-0.076	0.017	3.96E-06	0.074	0.041	0.069
17		rs72836424	C	T	21.624	-0.140	0.030	3.32E-06	0.036	0.064	0.578

18		rs73139629	A	C	21.555	-0.115	0.025	3.44E-06	0.013	0.070	0.857
1		rs113006825	T	C	19.937	-0.093	0.021	8.00E-06	0.063	0.049	0.196
2		rs12508214	C	T	21.026	-0.077	0.017	4.53E-06	0.060	0.043	0.158
3		rs138460696	A	G	19.451	0.139	0.032	1.03E-05	-0.085	0.076	0.265
4		rs1550196	G	A	24.849	0.131	0.026	6.20E-07	-0.032	0.068	0.638
5		rs2058609	A	G	21.837	0.082	0.017	2.97E-06	-0.036	0.045	0.419
6	**Ruminococcaceae**	rs2192926	A	G	21.290	-0.089	0.019	3.95E-06	0.023	0.043	0.586
7	**UCG009**	rs4079028	C	T	21.094	0.092	0.020	4.37E-06	0.070	0.047	0.135
8		rs4708333	T	G	23.122	-0.084	0.017	1.52E-06	0.042	0.043	0.323
9		rs6952765	G	A	19.269	0.073	0.017	1.14E-05	-0.078	0.043	0.070
10		rs758191	T	G	22.270	0.177	0.038	2.37E-06	-0.091	0.067	0.178
11		rs78410648	A	G	19.034	0.121	0.028	1.28E-05	-0.036	0.064	0.578
12		rs9558661	T	C	20.004	-0.090	0.020	7.73E-06	0.016	0.050	0.754
**Infertility**											
**Male infertility**											
1		rs12129908	C	A	20.285	0.089	0.020	6.67E-06	0.057	0.056	0.307
2	**Eubacterium**	rs12423772	G	T	22.834	0.141	0.030	1.77E-06	0.088	0.078	0.263
3	**(oxidoreducens**	rs2973294	G	T	22.331	0.092	0.020	2.29E-06	0.067	0.056	0.233
4	**group)**	rs34561138	G	A	22.087	0.216	0.046	2.61E-06	0.014	0.145	0.923
5		rs440215	C	T	22.812	0.093	0.020	1.79E-06	0.130	0.055	0.019
1		rs10248854	C	A	21.641	-0.053	0.011	3.29E-06	0.067	0.056	0.236
2		rs10797540	A	G	21.551	0.050	0.011	3.45E-06	-0.116	0.055	0.035
3		rs143694765	T	C	19.349	0.087	0.020	1.09E-05	-0.097	0.091	0.288
4	**Eubacterium**	rs2884897	A	G	20.034	-0.129	0.029	7.61E-06	0.177	0.151	0.241
5	**(rectale group)**	rs314726	T	C	23.329	0.053	0.011	1.37E-06	-0.056	0.055	0.314
6		rs35398954	A	G	26.653	-0.090	0.017	2.44E-07	0.152	0.075	0.043
7		rs59427698	A	G	19.379	-0.058	0.013	1.07E-05	0.088	0.071	0.217
8		rs62547233	A	G	19.908	0.054	0.012	8.12E-06	0.068	0.061	0.264
1		rs10417872	T	G	23.276	0.118	0.025	1.40E-06	0.077	0.061	0.205
2	**Lactococcus**	rs123059	T	C	24.769	-0.137	0.027	6.46E-07	0.009	0.067	0.892
3		rs12621813	G	A	20.413	0.108	0.024	6.24E-06	0.079	0.062	0.203

4		rs17168302	G	A	20.402	0.192	0.042	6.28E-06	0.184	0.090	0.040
5		rs2293361	C	T	21.369	-0.199	0.043	3.79E-06	-0.077	0.121	0.522
6		rs4766997	C	T	23.109	0.115	0.024	1.53E-06	-0.044	0.056	0.430
7		rs55910161	C	T	22.695	0.146	0.031	1.90E-06	0.076	0.088	0.391
8		rs6674304	C	T	20.619	0.201	0.044	5.60E-06	0.007	0.149	0.963
9		rs7992246	T	C	20.398	0.104	0.023	6.29E-06	0.046	0.056	0.413
**Abnormal spermatozoa**											
1		rs1007475	G	T	20.420	0.118	0.026	6.22E-06	0.089	0.053	0.092
2		rs11761679	T	C	23.170	0.155	0.032	1.48E-06	-0.038	0.067	0.572
3		rs142855850	A	G	20.113	0.205	0.046	7.30E-06	-0.032	0.081	0.692
4		rs16934069	T	C	19.961	-0.134	0.030	7.90E-06	-0.063	0.061	0.300
5		rs16941336	C	T	22.640	0.127	0.027	1.95E-06	0.144	0.058	0.012
6		rs17163238	G	A	20.802	0.141	0.031	5.09E-06	-0.008	0.060	0.889
7		rs28846706	A	G	20.159	0.176	0.039	7.13E-06	-0.052	0.081	0.520
8	**Butyrivibrio**	rs4537857	T	C	22.788	-0.125	0.026	1.81E-06	-0.029	0.051	0.566
9		rs486484	A	G	20.380	-0.108	0.024	6.35E-06	-0.013	0.049	0.787
10		rs4928024	A	G	20.137	-0.175	0.039	7.21E-06	0.016	0.064	0.801
11		rs72723662	C	T	24.869	0.224	0.045	6.14E-07	-0.012	0.072	0.869
12		rs74622183	A	G	22.040	-0.201	0.043	2.67E-06	-0.027	0.086	0.752
13		rs7752361	A	G	24.685	-0.119	0.024	6.75E-07	-0.076	0.048	0.113
14		rs7763512	G	A	22.374	0.120	0.025	2.24E-06	0.066	0.049	0.174
15		rs9349693	A	G	20.610	0.118	0.026	5.63E-06	0.052	0.052	0.321
1		rs12131224	C	T	20.424	0.117	0.026	6.20E-06	0.019	0.077	0.810
2	**Lachnospiraceae UCG001**	rs2050911	G	A	23.831	0.075	0.015	1.05E-06	0.008	0.051	0.882
3		rs2371284	T	C	20.056	-0.076	0.017	7.52E-06	0.065	0.057	0.259
4		rs437876	T	C	29.376	0.078	0.014	5.96E-08	0.038	0.051	0.456

5		rs4981345	T	C	20.717	-0.068	0.015	5.32E-06	-0.024	0.051	0.633
6		rs573933	T	C	21.565	-0.108	0.023	3.42E-06	-0.067	0.079	0.401
7		rs62496417	T	G	20.410	-0.075	0.017	6.25E-06	-0.051	0.058	0.380
8		rs7341608	T	C	19.514	-0.078	0.018	9.99E-06	-0.029	0.069	0.671
9		rs78848836	A	G	20.942	-0.119	0.026	4.74E-06	-0.030	0.078	0.698
10		rs8052586	T	C	19.841	0.176	0.040	8.42E-06	0.215	0.100	0.032
11		rs9403580	C	T	22.010	0.108	0.023	2.71E-06	0.032	0.071	0.653
12		rs985416	C	T	28.481	0.097	0.018	9.46E-08	0.113	0.062	0.067
13											
1		rs111509883	T	C	24.235	0.171	0.035	8.53E-07	-0.089	0.080	0.269
2		rs11685699	C	T	22.858	-0.141	0.030	1.74E-06	-0.049	0.091	0.591
3		rs117271932	A	G	22.326	0.208	0.044	2.30E-06	-0.109	0.104	0.292
4		rs12648235	T	C	19.565	0.079	0.018	9.72E-06	0.011	0.057	0.854
5		rs1304512	G	A	21.078	0.076	0.017	4.41E-06	-0.130	0.055	0.017
6		rs2104588	T	C	19.716	0.106	0.024	8.98E-06	-0.091	0.102	0.376
7		rs2495052	A	G	19.805	0.084	0.019	8.58E-06	0.018	0.069	0.796
8	**Prevotella 9**	rs2683313	A	G	22.843	-0.072	0.015	1.76E-06	-0.097	0.051	0.060
9		rs4968431	G	T	19.716	0.064	0.014	8.99E-06	-0.054	0.050	0.285
10		rs7237249	C	T	20.458	-0.082	0.018	6.10E-06	0.067	0.061	0.266
11		rs72815774	T	C	20.091	-0.176	0.039	7.38E-06	0.065	0.103	0.528
12		rs746764	T	C	22.457	-0.092	0.019	2.15E-06	-0.004	0.058	0.940
13		rs7976209	T	C	19.351	-0.087	0.020	1.09E-05	0.033	0.067	0.622
14		rs9428102	A	G	19.568	-0.078	0.018	9.71E-06	0.030	0.058	0.608
15		rs9613013	G	A	20.493	0.092	0.020	5.99E-06	-0.210	0.074	0.004
1	**Ruminococcaceae**	rs113006825	T	C	19.937	-0.093	0.021	8.00E-06	-0.059	0.058	0.309

2	**UCG009**	rs12508214	C	T	21.026	-0.077	0.017	4.53E-06	0.013	0.051	0.798
3		rs138460696	A	G	19.451	0.139	0.032	1.03E-05	0.033	0.092	0.717
4		rs1550196	G	A	24.849	0.131	0.026	6.20E-07	0.087	0.081	0.282
5		rs2058609	A	G	21.837	0.082	0.017	2.97E-06	-0.019	0.054	0.724
6		rs2192926	A	G	21.290	-0.089	0.019	3.95E-06	-0.014	0.051	0.787
7		rs4079028	C	T	21.094	0.092	0.020	4.37E-06	0.005	0.056	0.923
8		rs4708333	T	G	23.122	-0.084	0.017	1.52E-06	-0.065	0.051	0.202
9		rs6952765	G	A	19.269	0.073	0.017	1.14E-05	-0.040	0.052	0.441
10		rs758191	T	G	22.270	0.177	0.038	2.37E-06	0.174	0.082	0.033
11		rs78410648	A	G	19.034	0.121	0.028	1.28E-05	0.068	0.078	0.384
12		rs9558661	T	C	20.004	-0.090	0.020	7.73E-06	-0.046	0.061	0.452
1		rs10028567	C	T	23.047	-0.092	0.019	1.58E-06	-0.110	0.075	0.144
2		rs10448310	A	G	21.646	-0.052	0.011	3.28E-06	-0.030	0.050	0.551
3		rs11110281	T	C	36.572	-0.138	0.023	1.47E-09	-0.154	0.107	0.151
4		rs11720390	G	A	22.010	0.107	0.023	2.71E-06	0.181	0.102	0.075
5		rs17708276	A	G	21.652	-0.079	0.017	3.27E-06	0.109	0.080	0.177
6		rs1918540	G	A	21.659	0.060	0.013	3.26E-06	0.016	0.061	0.799
7	**Streptococcus**	rs2370083	G	T	19.317	-0.082	0.019	1.11E-05	-0.052	0.102	0.611
8		rs60486012	G	A	20.647	-0.091	0.020	5.52E-06	-0.219	0.124	0.078
9		rs6806351	T	C	21.515	-0.063	0.014	3.51E-06	-0.051	0.059	0.383
10		rs71481756	T	G	20.046	0.093	0.021	7.56E-06	0.000	0.101	1.000
11		rs7916711	A	G	22.407	0.103	0.022	2.21E-06	0.070	0.070	0.315
12		rs957755	T	G	23.544	-0.070	0.014	1.22E-06	-0.092	0.070	0.186
13		rs9895557	T	C	21.130	-0.051	0.011	4.29E-06	-0.012	0.048	0.801

Sperm Related Proteins

**Sperm acrosome membrane-associated protein 3 (SPACA3)**												
1	**Defluviitaleaceae UCG011**	rs112893842	T	C	23.899	0.114	0.023	1.02E-06	0.117	0.039	0.002
2		rs12500663	T	G	25.374	-0.115	0.023	4.72E-07	-0.029	0.035	0.407
3		rs1582238	T	C	23.167	0.081	0.017	1.49E-06	-0.002	0.026	0.955
4		rs2892880	G	A	20.258	0.082	0.018	6.77E-06	-0.002	0.029	0.933
5		rs4344384	G	T	20.981	0.072	0.016	4.64E-06	0.036	0.025	0.151
6		rs4677103	A	G	24.576	0.098	0.020	7.14E-07	-0.003	0.032	0.912
7		rs55658617	T	C	23.173	0.174	0.036	1.48E-06	0.042	0.045	0.355
8		rs9608282	T	G	22.733	0.143	0.030	1.86E-06	0.024	0.047	0.617
9		rs9725395	A	G	21.911	-0.138	0.030	2.86E-06	0.008	0.047	0.871
**Sperm acrosome associated 7 (SPACA7)**												
1	**Peptococcus**	rs10031059	T	C	28.784	-0.121	0.023	8.09E-08	-0.010	0.030	0.724
2		rs11001948	C	A	25.132	-0.191	0.038	5.35E-07	0.039	0.049	0.437
3		rs12069354	C	T	19.511	0.168	0.038	1.00E-05	-0.067	0.050	0.182
4		rs2054133	G	A	22.606	0.090	0.019	1.99E-06	0.028	0.025	0.257
5		rs36121075	A	G	21.094	-0.141	0.031	4.37E-06	-0.048	0.038	0.200
6		rs413827	G	A	21.537	0.110	0.024	3.47E-06	0.023	0.031	0.468
7		rs5770862	T	C	20.618	0.162	0.036	5.61E-06	0.084	0.047	0.074
8		rs62424012	G	A	22.045	0.137	0.029	2.66E-06	0.066	0.038	0.087
9		rs7033353	T	G	22.525	0.090	0.019	2.07E-06	0.009	0.026	0.708
10		rs72850165	T	C	19.985	-0.134	0.030	7.80E-06	-0.033	0.041	0.417
11		rs74592222	G	A	20.735	0.138	0.030	5.27E-06	0.027	0.040	0.501
12		rs77681628	C	T	26.745	0.200	0.039	2.32E-07	0.039	0.052	0.447
1	**Rikenellaceae RC9 (gut group)**	rs12501673	A	G	19.698	0.116	0.026	9.07E-06	-0.016	0.027	0.537
2		rs17032291	T	C	21.206	-0.170	0.037	4.13E-06	0.013	0.037	0.724

3		rs17582787	A	G	21.534	-0.158	0.034	3.48E-06	-0.010	0.036	0.776
4		rs2074881	T	C	19.283	-0.142	0.032	1.13E-05	0.059	0.033	0.071
5		rs2900503	G	T	27.829	-0.172	0.033	1.33E-07	0.005	0.035	0.891
6		rs2998141	T	C	21.644	-0.136	0.029	3.28E-06	-0.012	0.031	0.708
7		rs4270579	G	A	18.960	-0.118	0.027	1.33E-05	-0.013	0.028	0.631
8		rs4717843	G	T	20.992	-0.119	0.026	4.61E-06	0.078	0.026	0.003
9		rs7712231	A	G	19.886	0.156	0.035	8.22E-06	-0.041	0.035	0.245
10		rs80309088	G	A	20.606	0.174	0.038	5.64E-06	-0.032	0.038	0.398
11		rs9887954	G	A	21.272	-0.115	0.025	3.98E-06	0.016	0.025	0.525
**Sperm-associated antigen 11A (SPAG11A)**											
1		rs10502061	A	G	18.944	0.084	0.019	1.35E-05	-0.004	0.045	0.933
2		rs2014785	T	C	21.125	0.052	0.011	4.30E-06	0.019	0.026	0.468
3		rs2396460	T	C	21.897	-0.051	0.011	2.88E-06	0.004	0.025	0.871
4		rs2804244	A	G	22.882	-0.053	0.011	1.72E-06	0.018	0.025	0.468
5		rs3900776	G	A	21.675	-0.110	0.024	3.23E-06	0.061	0.047	0.195
6	**Anaerostipes**	rs62157625	T	C	22.787	0.089	0.019	1.81E-06	-0.043	0.039	0.269
7		rs62215703	G	A	22.260	0.064	0.014	2.38E-06	-0.066	0.030	0.027
8		rs6474958	A	G	19.935	-0.050	0.011	8.01E-06	0.013	0.025	0.603
9		rs6726833	C	A	21.463	-0.088	0.019	3.61E-06	-0.007	0.046	0.871
10		rs6854026	T	C	21.732	-0.051	0.011	3.14E-06	0.009	0.025	0.724
11		rs7193624	C	T	24.803	0.075	0.015	6.35E-07	-0.034	0.035	0.339
12		rs78735375	A	C	20.262	-0.137	0.031	6.75E-06	0.006	0.054	0.912
1		rs10464997	G	A	21.815	0.138	0.029	3.00E-06	0.039	0.036	0.275
2	**Oxalobacter**	rs11108500	A	G	21.708	-0.199	0.043	3.17E-06	-0.028	0.049	0.562
3		rs111966731	T	C	20.419	0.213	0.047	6.22E-06	0.017	0.053	0.741

4		rs12002250	A	C	21.679	0.217	0.047	3.22E-06	0.012	0.053	0.813
5		rs1569853	T	C	21.617	-0.138	0.030	3.33E-06	-0.049	0.033	0.138
6		rs36057338	G	T	24.323	0.208	0.042	8.15E-07	0.012	0.047	0.794
7		rs3862635	C	T	19.086	-0.172	0.039	1.25E-05	0.012	0.049	0.794
8		rs4428215	G	A	28.931	0.130	0.024	7.50E-08	0.059	0.030	0.048
9		rs6000536	C	T	26.637	-0.131	0.025	2.45E-07	-0.015	0.032	0.646
10		rs6993398	G	A	20.813	0.127	0.028	5.06E-06	-0.017	0.036	0.646
11		rs736744	C	T	31.135	0.118	0.021	2.41E-08	0.027	0.025	0.282
**Sperm-associated antigen 11B**		**(SPAG11B)**									
1		rs10150232	A	G	20.622	0.057	0.012	5.59E-06	0.047	0.029	0.105
2		rs11018566	A	G	18.272	-0.156	0.037	1.91E-05	-0.039	0.060	0.525
3		rs115414803	A	C	20.669	-0.144	0.032	5.46E-06	-0.099	0.062	0.110
4		rs1272208	G	T	22.202	-0.061	0.013	2.45E-06	-0.017	0.031	0.575
5		rs1431492	C	T	20.075	-0.065	0.015	7.45E-06	-0.014	0.033	0.661
6		rs17734739	T	C	19.603	0.066	0.015	9.53E-06	0.038	0.031	0.219
7	**Anaerotruncus**	rs34449434	A	C	19.208	-0.050	0.011	1.17E-05	0.056	0.029	0.054
8		rs4669806	G	T	21.962	0.058	0.012	2.78E-06	0.046	0.028	0.098
9		rs6494922	A	G	19.938	0.090	0.020	8.00E-06	-0.002	0.046	0.955
10		rs6563550	T	C	24.629	0.088	0.018	6.95E-07	0.075	0.045	0.093
11		rs7155595	C	A	20.575	0.054	0.012	5.73E-06	0.038	0.026	0.151
12		rs8005030	C	T	22.132	0.055	0.012	2.54E-06	0.032	0.027	0.229
13		rs9347879	T	C	20.988	0.051	0.011	4.62E-06	0.012	0.025	0.631
1		rs11149971	C	T	25.281	0.118	0.023	4.96E-07	-0.047	0.053	0.380
2	**Blautia**	rs113271346	C	T	20.679	0.078	0.017	5.43E-06	0.070	0.040	0.083
3		rs115043014	G	A	22.057	-0.207	0.044	2.65E-06	-0.063	0.060	0.288

4		rs117001700	T	C	19.826	0.196	0.044	8.48E-06	0.032	0.058	0.575
5		rs12453000	C	T	23.143	0.063	0.013	1.50E-06	0.031	0.031	0.316
6		rs16892041	T	C	19.277	-0.062	0.014	1.13E-05	0.030	0.035	0.380
7		rs2788271	T	G	18.592	-0.058	0.013	1.62E-05	0.000	0.033	1.000
8		rs3005511	A	G	20.464	0.050	0.011	6.08E-06	0.052	0.026	0.048
9		rs4926264	T	C	21.487	0.083	0.018	3.56E-06	0.040	0.048	0.407
10		rs67794373	C	T	23.760	0.060	0.012	1.09E-06	0.027	0.030	0.355
11		rs682885	A	G	21.119	-0.049	0.011	4.32E-06	-0.022	0.026	0.380
12		rs72973581	A	G	22.242	0.125	0.027	2.40E-06	0.018	0.053	0.741
13		rs7860714	A	G	20.897	-0.050	0.011	4.85E-06	-0.023	0.027	0.398
1	**Candidatus_Solea ferrea**	rs10090365	A	G	21.255	-0.083	0.018	4.02E-06	0.043	0.025	0.085
2		rs10108780	A	G	21.587	-0.093	0.020	3.38E-06	0.025	0.027	0.355
3		rs10809135	T	C	20.944	0.083	0.018	4.73E-06	0.001	0.025	0.977
4		rs4678258	T	C	20.912	0.099	0.022	4.81E-06	-0.027	0.030	0.372
5		rs61825792	T	C	23.418	0.112	0.023	1.30E-06	-0.025	0.032	0.427
6		rs6489992	A	G	20.192	-0.084	0.019	7.00E-06	0.034	0.025	0.178
7		rs6494306	A	G	20.474	-0.097	0.021	6.05E-06	0.030	0.030	0.316
8		rs7400877	T	C	19.976	-0.095	0.021	7.84E-06	0.048	0.029	0.098
9		rs9973954	A	G	20.842	0.089	0.020	4.99E-06	-0.009	0.028	0.759
1	**Coprococcus 2**	rs12634070	T	C	19.942	0.074	0.016	7.98E-06	-0.014	0.031	0.661
2		rs2482516	C	T	21.002	0.075	0.016	4.59E-06	-0.058	0.031	0.059
3		rs35890118	A	G	20.304	-0.067	0.015	6.61E-06	0.016	0.028	0.562
4		rs61823518	A	C	19.613	-0.096	0.022	9.48E-06	0.002	0.041	0.955
5		rs6677933	C	T	23.995	-0.080	0.016	9.66E-07	0.003	0.031	0.933
6		rs6894272	T	C	20.110	-0.113	0.025	7.31E-06	-0.023	0.046	0.617

7		rs72680320	T	C	21.766	-0.065	0.014	3.08E-06	0.066	0.025	0.009
8		rs9426473	A	G	20.244	0.073	0.016	6.82E-06	-0.027	0.031	0.380
9		rs12634070	T	C	19.942	0.074	0.016	7.98E-06	-0.014	0.031	0.661
1		rs12151423	A	G	19.869	0.101	0.023	8.29E-06	0.001	0.025	0.977
2		rs13139592	T	C	19.910	-0.146	0.033	8.12E-06	-0.024	0.036	0.501
3		rs1384962	A	G	20.613	0.121	0.027	5.62E-06	0.008	0.030	0.794
4		rs2913110	C	T	21.004	0.105	0.023	4.58E-06	0.014	0.025	0.589
5	**Eubacterium (brachy group)**	rs4862235	G	A	21.553	0.105	0.023	3.44E-06	0.012	0.025	0.646
6		rs55932844	A	G	22.357	-0.171	0.036	2.26E-06	-0.003	0.042	0.933
7		rs62348779	T	C	21.666	-0.201	0.043	3.25E-06	-0.051	0.047	0.288
8		rs6591893	G	A	20.281	0.108	0.024	6.69E-06	0.019	0.027	0.479
9		rs720439	A	G	19.845	-0.112	0.025	8.40E-06	-0.063	0.029	0.028
10		rs73199919	T	C	19.848	-0.237	0.053	8.38E-06	-0.046	0.051	0.363
**Spermatogenesis-associated protein 9 (SPATA9)**											
1		rs11150408	T	G	20.042	0.049	0.011	7.58E-06	0.002	0.025	0.933
2		rs12537781	T	C	19.675	-0.056	0.013	9.18E-06	0.067	0.029	0.021
3		rs13279148	G	A	22.477	0.072	0.015	2.13E-06	-0.048	0.034	0.151
4		rs1899291	C	T	21.523	0.070	0.015	3.50E-06	0.003	0.036	0.933
5		rs3005511	A	G	20.917	0.052	0.011	4.80E-06	-0.002	0.026	0.955
6	**Dorea**	rs345219	T	G	19.504	-0.050	0.011	1.00E-05	0.013	0.027	0.631
7		rs3752849	G	A	20.032	0.164	0.037	7.62E-06	-0.098	0.060	0.102
8		rs4793307	C	T	21.979	0.057	0.012	2.76E-06	-0.020	0.027	0.457
9		rs62503162	A	G	25.113	-0.097	0.019	5.41E-07	0.080	0.043	0.066
10		rs73729431	C	T	20.994	-0.137	0.030	4.61E-06	-0.015	0.053	0.776
1	**Lactobacillus**	rs11674854	C	T	23.338	-0.085	0.018	1.36E-06	0.017	0.026	0.525

2		rs12693845	C	T	20.608	-0.081	0.018	5.64E-06	0.059	0.026	0.020
3		rs1530559	G	A	20.355	0.080	0.018	6.43E-06	-0.027	0.025	0.282
4		rs16861661	G	A	23.049	-0.183	0.038	1.58E-06	-0.013	0.049	0.794
5		rs62314653	C	A	22.626	0.188	0.039	1.97E-06	-0.069	0.056	0.219
6		rs7399658	G	A	23.313	-0.107	0.022	1.38E-06	-0.040	0.030	0.186
7		rs75127669	C	A	20.278	0.140	0.031	6.70E-06	-0.152	0.048	0.001
8		rs768253	T	G	21.252	-0.079	0.017	4.03E-06	0.009	0.026	0.741
9		rs77478751	A	G	21.361	-0.220	0.048	3.80E-06	0.160	0.052	0.002
10		rs921925	A	C	23.495	0.099	0.020	1.25E-06	0.019	0.030	0.525
1		rs10279978	A	G	23.749	-0.062	0.013	1.10E-06	-0.025	0.027	0.355
2		rs11221428	T	C	21.088	-0.073	0.016	4.39E-06	-0.013	0.032	0.692
3		rs16843578	C	T	19.785	-0.088	0.020	8.67E-06	-0.025	0.041	0.550
4		rs28603357	T	C	20.493	-0.215	0.047	5.98E-06	0.061	0.063	0.331
5		rs34302036	A	G	20.737	0.055	0.012	5.27E-06	0.011	0.025	0.661
6		rs61841503	G	A	29.351	0.093	0.017	6.04E-08	0.070	0.034	0.039
7	**Romboutsia**	rs62504452	A	G	20.544	-0.071	0.016	5.83E-06	0.042	0.034	0.214
8		rs7109293	A	G	19.957	0.092	0.021	7.92E-06	0.010	0.048	0.832
9		rs75200530	T	G	20.500	-0.191	0.042	5.96E-06	-0.096	0.056	0.085
10		rs75987356	G	A	21.347	-0.130	0.028	3.83E-06	-0.052	0.049	0.295
11		rs77702691	A	G	20.493	-0.094	0.021	5.99E-06	-0.050	0.042	0.229
12		rs9389266	T	G	19.821	0.072	0.016	8.50E-06	0.034	0.035	0.331
13		rs9567264	C	T	20.697	0.058	0.013	5.38E-06	0.010	0.028	0.708
1		rs10916131	C	T	22.321	-0.069	0.015	2.31E-06	0.038	0.034	0.263
2	**Ruminococcaceae UCG002**	rs10927423	C	A	23.340	-0.071	0.015	1.36E-06	-0.005	0.034	0.891
3		rs10964441	G	A	18.683	-0.149	0.034	1.54E-05	0.075	0.057	0.191

4		rs113147300	A	G	21.240	-0.076	0.016	4.05E-06	0.028	0.036	0.437
5		rs11607472	A	G	19.580	-0.078	0.018	9.65E-06	0.035	0.040	0.372
6		rs116974815	C	A	22.890	-0.190	0.040	1.72E-06	0.112	0.057	0.048
7		rs12463378	A	G	21.694	-0.052	0.011	3.20E-06	0.028	0.026	0.282
8		rs15256	C	T	18.928	0.073	0.017	1.36E-05	0.051	0.043	0.234
9		rs55793120	T	C	25.119	0.137	0.027	5.39E-07	-0.058	0.052	0.269
10		rs56030423	G	A	20.640	-0.098	0.022	5.54E-06	-0.038	0.046	0.407
11		rs57079348	T	G	19.632	-0.077	0.017	9.39E-06	-0.040	0.041	0.324
12		rs62374283	T	C	23.215	-0.058	0.012	1.45E-06	-0.009	0.028	0.741
13		rs6542556	A	G	19.972	0.051	0.011	7.86E-06	0.005	0.026	0.832
14		rs6793778	C	T	19.896	-0.056	0.013	8.18E-06	0.002	0.029	0.955
15		rs7120052	A	C	21.254	0.062	0.014	4.02E-06	-0.014	0.034	0.676
16		rs7155595	C	A	23.734	0.057	0.012	1.11E-06	-0.063	0.026	0.016
17		rs7249614	A	G	19.777	-0.049	0.011	8.70E-06	0.006	0.027	0.813
18		rs7342369	C	A	20.694	-0.053	0.012	5.39E-06	0.020	0.027	0.457
19		rs76847269	A	G	21.077	0.164	0.036	4.41E-06	-0.046	0.056	0.417
20		rs77564310	A	C	25.630	-0.071	0.014	4.14E-07	-0.014	0.032	0.676
21		rs79016051	C	T	21.964	-0.089	0.019	2.78E-06	-0.027	0.043	0.537
22		rs882348	A	G	20.057	-0.080	0.018	7.52E-06	-0.013	0.042	0.759
1	**Ruminococcaceae UCG014**	rs10495392	C	T	19.417	-0.082	0.019	1.05E-05	-0.066	0.038	0.085
2		rs10791168	A	G	19.614	-0.066	0.015	9.48E-06	-0.021	0.035	0.550
3		rs10941294	C	T	22.036	-0.122	0.026	2.68E-06	-0.053	0.049	0.282
4		rs115777838	T	C	23.731	-0.188	0.039	1.11E-06	0.059	0.059	0.316
5		rs12638134	T	G	23.702	0.058	0.012	1.12E-06	-0.022	0.025	0.380
6		rs34402072	C	T	19.431	-0.069	0.016	1.04E-05	-0.053	0.032	0.100

7		rs56105232	G	A	21.678	0.139	0.030	3.22E-06	0.013	0.054	0.813
8		rs72809222	T	C	23.078	0.067	0.014	1.56E-06	0.016	0.028	0.562
9		rs73186226	G	A	21.011	-0.099	0.022	4.57E-06	-0.090	0.044	0.041
10		rs853612	A	G	19.585	-0.053	0.012	9.62E-06	-0.034	0.026	0.186
11		rs995642	C	T	22.562	0.060	0.013	2.03E-06	0.046	0.027	0.087
**Spermatogenesis-associated protein 20 (SPATA20)**											
1		rs10065321	T	C	22.503	-0.051	0.011	2.10E-06	-0.007	0.025	0.794
2		rs10497836	C	T	19.493	-0.052	0.012	1.01E-05	-0.046	0.028	0.102
3		rs1667315	G	A	20.374	0.049	0.011	6.37E-06	0.023	0.025	0.363
4		rs2114677	C	T	20.368	-0.104	0.023	6.39E-06	-0.038	0.057	0.501
5		rs2171249	C	T	20.950	0.107	0.023	4.71E-06	0.051	0.045	0.263
6	**Subdoligranulum**	rs35940633	G	A	21.577	-0.051	0.011	3.40E-06	-0.029	0.027	0.269
7		rs3761728	T	G	20.903	-0.054	0.012	4.83E-06	-0.009	0.028	0.741
8		rs4347804	A	G	21.579	0.166	0.036	3.40E-06	-0.035	0.062	0.575
9		rs6555306	T	C	22.705	-0.074	0.016	1.89E-06	-0.048	0.037	0.200
10		rs75158211	T	C	20.616	-0.072	0.016	5.61E-06	-0.031	0.038	0.407
11		rs76528319	G	T	21.271	-0.143	0.031	3.99E-06	-0.007	0.062	0.912
1		rs11054680	T	C	21.295	-0.105	0.023	3.94E-06	-0.028	0.038	0.468
2		rs11666533	C	T	20.211	-0.112	0.025	6.93E-06	-0.049	0.041	0.234
3		rs12603364	T	C	24.148	0.111	0.023	8.92E-07	0.012	0.037	0.741
4	**Turicibacter**	rs149744580	A	G	28.997	0.170	0.032	7.25E-08	0.082	0.052	0.117
5		rs2834977	T	C	21.248	-0.096	0.021	4.03E-06	-0.028	0.035	0.417
6		rs2952020	G	A	20.967	-0.076	0.017	4.67E-06	-0.052	0.028	0.058
7		rs4869133	G	A	23.267	0.131	0.027	1.41E-06	0.064	0.046	0.158
8		rs55756211	T	C	22.871	-0.115	0.024	1.73E-06	0.019	0.040	0.631

9		rs7199484	G	A	20.853	-0.073	0.016	4.96E-06	0.034	0.027	0.209
**Zona pellucida sperm-binding protein 4 (ZPBP4)**											
1	**Adlercreutzia**	rs11604400	C	T	19.060	-0.103	0.023	1.27E-05	0.056	0.041	0.170
2		rs13231526	C	A	21.123	0.143	0.031	4.31E-06	-0.052	0.048	0.275
3		rs34181676	G	T	22.867	-0.135	0.028	1.74E-06	0.029	0.044	0.513
4		rs55719207	G	A	19.577	-0.070	0.016	9.66E-06	0.003	0.025	0.912
5		rs6664405	T	C	20.451	-0.095	0.021	6.12E-06	0.018	0.035	0.603
6		rs7680684	C	T	24.371	-0.083	0.017	7.95E-07	0.076	0.027	0.005
7		rs9490822	C	T	22.229	-0.073	0.016	2.42E-06	0.020	0.025	0.447
8		rs9915817	T	C	19.810	0.075	0.017	8.55E-06	-0.021	0.027	0.437
1	**Lachnoclostridium**	rs1031599	G	T	20.039	-0.079	0.018	7.59E-06	-0.006	0.044	0.891
2		rs12566975	T	C	19.580	-0.047	0.011	9.65E-06	0.020	0.025	0.427
3		rs1528479	G	A	19.783	-0.050	0.011	8.67E-06	-0.010	0.027	0.708
4		rs1997204	T	C	19.941	-0.108	0.024	7.99E-06	0.082	0.053	0.126
5		rs2385421	A	G	17.046	0.075	0.018	3.65E-05	-0.008	0.045	0.851
6		rs3821998	C	A	20.144	-0.086	0.019	7.18E-06	0.098	0.047	0.036
7		rs4738679	G	A	20.813	-0.052	0.011	5.06E-06	0.013	0.026	0.631
8		rs6112314	A	C	26.964	-0.056	0.011	2.07E-07	-0.028	0.025	0.263
9		rs615997	T	C	23.094	0.051	0.011	1.54E-06	-0.051	0.025	0.042
10		rs62285313	A	G	22.655	0.086	0.018	1.94E-06	-0.056	0.040	0.166
11		rs72829893	G	T	19.199	0.117	0.027	1.18E-05	-0.031	0.057	0.589
12		rs78068103	A	G	20.814	0.089	0.019	5.06E-06	-0.007	0.050	0.891
13		rs789029	C	T	21.603	-0.064	0.014	3.35E-06	0.014	0.035	0.692
1	**Lachnospiraceae (ND3007 group)**	rs2861203	G	A	20.218	0.057	0.013	6.91E-06	0.039	0.030	0.191
2		rs72776675	T	C	19.123	-0.065	0.015	1.23E-05	-0.027	0.032	0.398

3		rs9932954	A	G	23.467	-0.056	0.012	1.27E-06	-0.040	0.025	0.110
1		rs10916131	C	T	22.321	-0.069	0.015	2.31E-06	0.023	0.034	0.490
2		rs10927423	C	A	23.340	-0.071	0.015	1.36E-06	-0.015	0.034	0.646
3		rs10964441	G	A	18.683	-0.149	0.034	1.54E-05	0.072	0.057	0.204
4		rs113147300	A	G	21.240	-0.076	0.016	4.05E-06	-0.026	0.036	0.479
5		rs11607472	A	G	19.580	-0.078	0.018	9.65E-06	0.061	0.040	0.123
6		rs116974815	C	A	22.890	-0.190	0.040	1.72E-06	-0.047	0.057	0.398
7		rs12463378	A	G	21.694	-0.052	0.011	3.20E-06	0.023	0.026	0.363
8		rs15256	C	T	18.928	0.073	0.017	1.36E-05	-0.025	0.043	0.562
9		rs55793120	T	C	25.119	0.137	0.027	5.39E-07	-0.102	0.052	0.051
10		rs56030423	G	A	20.640	-0.098	0.022	5.54E-06	0.029	0.046	0.525
11	**Ruminococcaceae**	rs57079348	T	G	19.632	-0.077	0.017	9.39E-06	0.043	0.041	0.288
12	**UCG002**	rs62374283	T	C	23.215	-0.058	0.012	1.45E-06	-0.061	0.028	0.028
13		rs6542556	A	G	19.972	0.051	0.011	7.86E-06	-0.016	0.026	0.537
14		rs6793778	C	T	19.896	-0.056	0.013	8.18E-06	-0.004	0.029	0.891
15		rs7120052	A	C	21.254	0.062	0.014	4.02E-06	-0.049	0.034	0.145
16		rs7155595	C	A	23.734	0.057	0.012	1.11E-06	-0.005	0.026	0.832
17		rs7249614	A	G	19.777	-0.049	0.011	8.70E-06	0.025	0.027	0.339
18		rs7342369	C	A	20.694	-0.053	0.012	5.39E-06	0.033	0.027	0.214
19		rs76847269	A	G	21.077	0.164	0.036	4.41E-06	-0.073	0.056	0.191
20		rs77564310	A	C	25.630	-0.071	0.014	4.14E-07	0.028	0.032	0.372
21		rs79016051	C	T	21.964	-0.089	0.019	2.78E-06	0.036	0.043	0.398
22		rs882348	A	G	20.057	-0.080	0.018	7.52E-06	-0.021	0.042	0.631
1	**Ruminococcus**	rs10931481	G	A	21.861	0.061	0.013	2.93E-06	0.021	0.027	0.427
2	**(gauvreauii**	rs11750752	C	T	20.606	0.071	0.016	5.64E-06	0.060	0.031	0.056

3	**group）**	rs12079579	A	G	20.034	0.096	0.021	7.61E-06	-0.038	0.045	0.398
4		rs1391597	C	T	22.345	0.059	0.012	2.28E-06	0.023	0.026	0.372
5		rs2047242	A	G	25.552	-0.068	0.013	4.31E-07	-0.052	0.027	0.052
6		rs2105937	A	G	20.610	0.058	0.013	5.63E-06	0.006	0.026	0.813
7		rs2166943	A	C	21.077	0.057	0.012	4.41E-06	0.030	0.025	0.234
8		rs289410	G	A	22.168	-0.065	0.014	2.50E-06	-0.020	0.029	0.490
9		rs431418	A	G	20.321	-0.095	0.021	6.55E-06	-0.024	0.041	0.562
10		rs71386687	T	G	25.733	0.121	0.024	3.92E-07	0.016	0.047	0.724
11		rs73802842	C	A	18.859	0.074	0.017	1.41E-05	0.024	0.035	0.490
12		rs9870933	A	G	24.313	0.062	0.013	8.19E-07	0.042	0.025	0.098
1		rs11054680	T	C	21.295	-0.105	0.023	3.94E-06	0.021	0.038	0.589
2		rs11666533	C	T	20.211	-0.112	0.025	6.93E-06	-0.009	0.041	0.832
3		rs12603364	T	C	24.148	0.111	0.023	8.92E-07	0.084	0.037	0.024
4		rs149744580	A	G	28.997	0.170	0.032	7.25E-08	0.082	0.052	0.115
5	**Turicibacter**	rs2834977	T	C	21.248	-0.096	0.021	4.03E-06	-0.037	0.035	0.288
6		rs2952020	G	A	20.967	-0.076	0.017	4.67E-06	-0.010	0.028	0.724
7		rs4869133	G	A	23.267	0.131	0.027	1.41E-06	0.090	0.046	0.049
8		rs55756211	T	C	22.871	-0.115	0.024	1.73E-06	-0.002	0.040	0.955
9		rs7199484	G	A	20.853	-0.073	0.016	4.96E-06	0.007	0.027	0.794

Table S3, Supplemental Digital Content. MR estimates for the association between gut microbiota and prostatitis

**Bacterial genus(exposure)**	**No. of SNP**	**MR method**	**OR**	**95% CI**	**P-value**
**Erysipelatoclostridium**	15	MR Egger	0.99	[0.35 - 2.78]	0.98
		Weighted median	0.75	[0.53 - 1.08]	0.12
		IVW	0.71	[0.55 - 0.92]	0.01
		Simple mode	0.77	[0.43 - 1.38]	0.39
		Weighted mode	0.76	[0.43 - 1.34]	0.36
**Faecalibacterium**	10	MR Egger	2.58	[1.29 - 5.18]	0.03
		Weighted median	1.54	[0.95 - 2.50]	0.08
		IVW	1.59	[1.08 - 2.34]	0.02
		Simple mode	1.64	[0.75 - 3.61]	0.25
		Weighted mode	1.64	[0.82 - 3.31]	0.20
**Lachnospiraceae UCG004**	12	MR Egger	0.91	[0.20 - 4.02]	0.90
		Weighted median	1.89	[1.21 - 2.97]	0.01
		IVW	1.64	[1.15 - 2.34]	0.01
		Simple mode	2.21	[1.05 - 4.67]	0.06
		Weighted mode	2.20	[1.09 - 4.46]	0.05
**Odoribacter**	7	MR Egger	1.50	[0.26 - 8.67]	0.67
		Weighted median	2.05	[1.13 - 3.72]	0.02
		IVW	1.68	[1.01 - 2.81]	0.046
		Simple mode	2.22	[0.88 - 5.65]	0.14
		Weighted mode	2.20	[0.91 - 5.33]	0.13
**Paraprevotella**	13	MR Egger	2.07	[0.89 - 4.79]	0.12
		Weighted median	1.20	[0.90 - 1.61]	0.22
		IVW	1.28	[1.03 - 1.60]	0.03
		Simple mode	1.11	[0.64 - 1.93]	0.72
		Weighted mode	1.09	[0.61 - 1.96]	0.78
**Parasutterella**	14	MR Egger	0.71	[0.35 - 1.46]	0.37
		Weighted median	0.75	[0.53 - 1.07]	0.12

		IVW	0.74	[0.57 - 0.96]	0.02
		Simple mode	0.67	[0.37 - 1.22]	0.21
		Weighted mode	0.74	[0.46 - 1.19]	0.23
	12	MR Egger	1.33	[0.50 - 3.53]	0.58
		Weighted median	0.81	[0.58 - 1.12]	0.20
**Ruminococcaceae UCG009**		IVW	0.77	[0.60 - 0.98]	0.03
		Simple mode	0.82	[0.47 - 1.45]	0.51
		Weighted mode	0.84	[0.46 - 1.53]	0.58
	6	MR Egger	0.15	[0.02 - 1.33]	0.16
		Weighted median	0.70	[0.46 - 1.07]	0.10
**Slackia**		IVW	0.69	[0.49 - 0.96]	0.03
		Simple mode	0.73	[0.42 - 1.26]	0.31
		Weighted mode	0.73	[0.42 - 1.29]	0.33
	12	MR Egger	3.69	[0.89 - 15.36]	0.10
		Weighted median	1.63	[1.04 - 2.54]	0.03
**Sutterella**		IVW	1.58	[1.13 - 2.19]	0.01
		Simple mode	1.67	[0.74 - 3.73]	0.24
		Weighted mode	1.66	[0.72 - 3.82]	0.26

Table S4, Supplemental Digital Content. MR estimates for the association between gut microbiota and orchitis and epididymitis

Bacterial genus(exposure)	No. of SNP	MR method	OR	95% CI	P-value
**Eubacterium (ruminantium group)**	18	MR Egger	1.04	[0.48 - 2.28]	0.92
		Weighted median	0.78	[0.56 - 1.07]	0.13
		IVW	0.74	[0.58 - 0.93]	0.01
		Simple mode	0.82	[0.48 - 1.39]	0.47
		Weighted mode	0.86	[0.52 - 1.42]	0.56
**Ruminococcaceae UCG009**	12	MR Egger	0.76	[0.24 - 2.41]	0.65
		Weighted median	0.62	[0.43 - 0.90]	0.01
		IVW	0.68	[0.51 - 0.91]	0.01
		Simple mode	0.65	[0.38 - 1.13]	0.16
		Weighted mode	0.65	[0.37 - 1.12]	0.15

Table S5, Supplemental Digital Content. MR estimates for the association between gut microbiota and male infertility

Bacterial genus(exposure)	No. of SNP	MR method	OR	95% CI	P-value
**Eubacterium (oxidoreducens group)**	5	MR Egger	0.72	[0.10 - 5.24]	0.76
		Weighted median	1.90	[0.97 - 3.73]	0.06
		IVW	2.05	[1.20 - 3.49]	0.01
		Simple mode	1.93	[0.80 - 4.66]	0.22
		Weighted mode	1.92	[0.79 - 4.65]	0.22
**Eubacterium (rectale group)**	8	MR Egger	0.16	[0.01 - 2.35]	0.23
		Weighted median	0.27	[0.10 - 0.74]	0.01
		IVW	0.31	[0.15 - 0.64]	0.002
		Simple mode	0.28	[0.08 - 1.01]	0.09
		Weighted mode	0.29	[0.08 - 1.06]	0.10
**Lactococcus**	9	MR Egger	2.25	[0.46 - 11.06]	0.35
		Weighted median	1.56	[0.95 - 2.55]	0.08
		IVW	1.45	[1.01 - 2.06]	0.04
		Simple mode	1.73	[0.80 - 3.71]	0.20
		Weighted mode	1.77	[0.76 - 4.12]	0.22

Table S6, Supplemental Digital Content. MR estimates for the association between gut microbiota and abnormal spermatozoa

**Bacterial genus(exposure)**	**No. of SNP**	**MR method**	**OR**	**95% CI**	**P-value**
	15	MR Egger	0.40	[0.16 - 1.03]	0.08
		Weighted median	1.13	[0.84 - 1.52]	0.42
**Butyrivibrio**		IVW	1.24	[1.00 - 1.52]	0.047
		Simple mode	0.97	[0.58 - 1.62]	0.90
		Weighted mode	0.96	[0.60 - 1.52]	0.85
	13	MR Egger	4.14	[0.88 - 19.46]	0.10
		Weighted median	1.44	[0.85 - 2.44]	0.18
**Lachnospiraceae UCG001**		IVW	1.57	[1.07 - 2.32]	0.02
		Simple mode	1.39	[0.59 - 3.27]	0.46
		Weighted mode	1.36	[0.58 - 3.17]	0.49
	15	MR Egger	0.64	[0.19 - 2.10]	0.47
		Weighted median	0.67	[0.42 - 1.07]	0.09
**Prevotella 9**		IVW	0.67	[0.45 - 0.99]	0.046
		Simple mode	0.62	[0.29 - 1.33]	0.24
		Weighted mode	0.64	[0.34 - 1.20]	0.18
	12	MR Egger	5.10	[1.27 - 20.52]	0.04
		Weighted median	1.57	[0.98 - 2.50]	0.06
**Ruminococcaceae UCG009**		IVW	1.44	[1.02 - 2.04]	0.04
		Simple mode	1.78	[0.83 - 3.80]	0.17
		Weighted mode	1.84	[0.78 - 4.12]	0.16
	13	MR Egger	4.53	[0.79 - 25.99]	0.12
		Weighted median	2.05	[1.04 - 4.03]	0.04
**Streptococcus**		IVW	2.07	[1.24 - 3.44]	0.01
		Simple mode	1.95	[0.68 - 5.62]	0.24
		Weighted mode	2.14	[0.78 - 5.90]	0.17

Table S7, Supplemental Digital Content. MR estimates for the association between gut microbiota and sperm acrosome membrane-associated protein 3

Bacterial genus (exposure)	No. of SNP	MR method	β	95%CI	OR	95% CI	P-value
**Defluviitaleaceae UCG011**	9	MR Egger	0.27	[-0.57 - 1.11]	1.31	[0.57 - 3.04]	0.54
		Weighted median	0.17	[-0.11 - 0.46]	1.19	[0.90 - 1.58]	0.23
		IVW	0.23	[0.01 - 0.44]	1.25	[1.01 - 1.56]	0.04
		Simple mode	0.05	[-0.32 - 0.43]	1.05	[0.72 - 1.53]	0.80
		Weighted mode	0.09	[-0.28 - 0.45]	1.09	[0.76 - 1.57]	0.65

Table S8, Supplemental Digital Content. MR estimates for the association between gut microbiota and sperm acrosome associated 7

Bacterial genus(exposure)	No. of SNP	MR method	β	95%CI	OR	95% CI	P-value
**Peptococcus**	12	MR Egger	-0.06	[-0.68 - 0.57]	0.95	[0.51 - 1.77]	0.86
		Weighted median	0.20	[-0.01 - 0.41]	1.22	[0.99 - 1.50]	0.07
		IVW	0.17	[0.01 - 0.33]	1.18	[1.01 - 1.39]	0.03
		Simple mode	0.21	[-0.14 - 0.56]	1.23	[0.87 - 1.75]	0.27
		Weighted mode	0.20	[-0.14 - 0.54]	1.22	[0.87 - 1.72]	0.27
**Rikenellaceae RC9 (gut group)**	11	MR Egger	0.12	[-0.75 - 0.99]	1.13	[0.47 - 2.68]	0.80
		Weighted median	-0.12	[-0.31 - 0.06]	0.89	[0.74 - 1.06]	0.20
		IVW	-0.15	[-0.28 - -0.02]	0.86	[0.75 - 0.98]	0.03
		Simple mode	-0.10	[-0.39 - 0.18]	0.90	[0.68 - 1.20]	0.49
		Weighted mode	-0.09	[-0.35 - 0.16]	0.91	[0.70 - 1.18]	0.49

Table S9, Supplemental Digital Content. MR estimates for the association between gut microbiota and sperm-associated antigen 11A

Bacterial genus(exposure)	No. of SNP	MR method	β	95%CI	OR	95% CI	P-value
**Anaerostipes**	12	MR Egger	-0.40	[-1.28 - 0.47]	0.67	[0.28 - 1.60]	0.39
		Weighted median	-0.23	[-0.55 - 0.10]	0.80	[0.57 - 1.11]	0.18
		IVW	-0.27	[-0.54 - -0.005]	0.76	[0.59 - 1.00]	0.046
		Simple mode	-0.15	[-0.74 - 0.43]	0.86	[0.48 - 1.54]	0.62
		Weighted mode	-0.15	[-0.69 - 0.39]	0.86	[0.50 - 1.48]	0.60
**Oxalobacter**	11	MR Egger	-0.16	[-0.83 - 0.50]	0.85	[0.44 - 1.65]	0.64
		Weighted median	0.12	[-0.07 - 0.31]	1.13	[0.93 - 1.36]	0.22
		IVW	0.16	[0.01 - 0.30]	1.17	[1.01 - 1.35]	0.03
		Simple mode	0.10	[-0.21 - 0.40]	1.10	[0.81 - 1.50]	0.55
		Weighted mode	0.10	[-0.21 - 0.42]	1.11	[0.81 - 1.52]	0.53

Table S10, Supplemental Digital Content. MR estimates for the association between gut microbiota and perm-associated antigen 11B

Bacterial genus(exposure)	No. of SNP	MR method	β	95%CI	OR	95% CI	P-value
**Anaerotruncus**	13	MR Egger	0.58	[-0.26 - 1.41]	1.78	[0.77 - 4.10]	0.20
		Weighted median	0.52	[0.18 - 0.87]	1.69	[1.19 - 2.39]	0.003
		IVW	0.41	[0.15 - 0.67]	1.51	[1.16 - 1.96]	0.002
		Simple mode	0.66	[0.08 - 1.24]	1.94	[1.09 - 3.46]	0.04
		Weighted mode	0.62	[0.07 - 1.18]	1.87	[1.07 - 3.25]	0.05
**Blautia**	13	MR Egger	0.05	[-0.47 - 0.57]	1.05	[0.62 - 1.76]	0.87
		Weighted median	0.28	[-0.04 - 0.61]	1.33	[0.96 - 1.84]	0.08
		IVW	0.28	[0.04 - 0.52]	1.33	[1.04 - 1.69]	0.02
		Simple mode	0.39	[-0.12 - 0.89]	1.47	[0.88 - 2.44]	0.16
		Weighted mode	0.30	[-0.13 - 0.73]	1.35	[0.88 - 2.08]	0.20
**Candidatus_Soleaferrea**	9	MR Egger	-0.23	[-2.36 - 1.91]	0.80	[0.09 - 6.75]	0.84
		Weighted median	-0.27	[-0.53 - -0.02]	0.76	[0.59 - 0.98]	0.04
		IVW	-0.29	[-0.48 - -0.09]	0.75	[0.62 - 0.91]	0.004
		Simple mode	-0.29	[-0.68 - 0.10]	0.75	[0.51 - 1.11]	0.19
		Weighted mode	-0.28	[-0.67 - 0.11]	0.76	[0.51 - 1.12]	0.20
**Coprococcus 2**	9	MR Egger	1.47	[-0.29 - 3.24]	4.35	[0.75 - 25.41]	0.15
		Weighted median	-0.20	[-0.59 - 0.19]	0.82	[0.55 - 1.21]	0.32
		IVW	-0.31	[-0.60 - -0.02]	0.73	[0.55 - 0.98]	0.03
		Simple mode	-0.14	[-0.75 - 0.47]	0.87	[0.47 - 1.60]	0.66
		Weighted mode	-0.12	[-0.69 - 0.44]	0.88	[0.50 - 1.55]	0.68
**Eubacterium (brachy group)**	13	MR Egger	0.20	[-0.37 - 0.78]	1.22	[0.69 - 2.17]	0.51
		Weighted median	0.15	[-0.04 - 0.33]	1.16	[0.96 - 1.40]	0.13
		IVW	0.17	[0.02 - 0.32]	1.18	[1.02 - 1.37]	0.03
		Simple mode	0.15	[-0.12 - 0.42]	1.16	[0.89 - 1.53]	0.30
		Weighted mode	0.16	[-0.11 - 0.42]	1.17	[0.90 - 1.52]	0.27

Table S11, Supplemental Digital Content. MR estimates for the association between gut microbiota and spermatogenesis-associated protein 9

Bacterial genus (exposure)	No. of SNP	MR method	β	95%CI	OR	95% CI	P-value
**Dorea**	10	MR Egger	-0.46	[-1.23 - 0.32]	0.63	[0.29 - 1.37]	0.28
		Weighted median	-0.31	[-0.70 - 0.09]	0.74	[0.50 - 1.09]	0.13
		IVW	-0.37	[-0.66 - -0.09]	0.69	[0.52 - 0.91]	0.01
		Simple mode	-0.07	[-0.78 - 0.63]	0.93	[0.46 - 1.88]	0.84
		Weighted mode	-0.05	[-0.72 - 0.62]	0.96	[0.49 - 1.87]	0.90
**Lactobacillus**	10	MR Egger	-0.54	[-1.39 - 0.31]	0.58	[0.25 - 1.36]	0.24
		Weighted median	-0.20	[-0.50 - 0.10]	0.82	[0.60 - 1.11]	0.20
		IVW	-0.28	[-0.57 - -0.004]	0.75	[0.57 - 1.00]	0.05
		Simple mode	-0.17	[-0.75 - 0.40]	0.84	[0.47 - 1.50]	0.57
		Weighted mode	-0.12	[-0.65 - 0.41]	0.89	[0.52 - 1.50]	0.67
**Romboutsia**	13	MR Egger	0.19	[-0.37 - 0.75]	1.21	[0.69 - 2.12]	0.52
		Weighted median	0.32	[0.01 - 0.63]	1.38	[1.01 - 1.89]	0.04
		IVW	0.24	[0.03 - 0.46]	1.28	[1.03 - 1.59]	0.03
		Simple mode	0.34	[-0.18 - 0.85]	1.40	[0.84 - 2.35]	0.23
		Weighted mode	0.41	[-0.15 - 0.97]	1.51	[0.86 - 2.65]	0.17
**Ruminococcaceae UCG002**	22	MR Egger	-0.39	[-0.90 - 0.13]	0.68	[0.40 - 1.14]	0.16
		Weighted median	-0.28	[-0.55 - -0.003]	0.76	[0.58 - 1.00]	0.05
		IVW	-0.20	[-0.39 - -0.01]	0.82	[0.68 - 0.99]	0.04
		Simple mode	-0.38	[-0.93 - 0.17]	0.68	[0.39 - 1.18]	0.19
		Weighted mode	-0.41	[-0.92 - 0.09]	0.66	[0.40 - 1.09]	0.12
**Ruminococcaceae UCG014**	11	MR Egger	-0.12	[-0.87 - 0.64]	0.89	[0.42 - 1.89]	0.77
		Weighted median	0.30	[-0.05 - 0.66]	1.36	[0.95 - 1.94]	0.10
		IVW	0.31	[0.03 - 0.59]	1.36	[1.03 - 1.80]	0.03
		Simple mode	0.67	[-0.02 - 1.37]	1.96	[0.98 - 3.92]	0.09
		Weighted mode	0.60	[-0.16 - 1.35]	1.82	[0.86 - 3.86]	0.15

Table S12, Supplemental Digital Content. MR estimates for the association between gut microbiota and spermatogenesis-associated protein 20

Bacterial genus (exposure)	No. of SNP	MR method	β	95%CI	OR	95% CI	P-value
**Subdoligranulum**	11	MR Egger	-0.11	[-0.85 - 0.63]	0.89	[0.43 - 1.87]	0.77
		Weighted median	0.39	[0.02 - 0.75]	1.47	[1.02 - 2.12]	0.04
		IVW	0.31	[0.03 - 0.59]	1.36	[1.03 - 1.81]	0.03
		Simple mode	0.45	[-0.19 - 1.09]	1.57	[0.83 - 2.99]	0.20
		Weighted mode	0.45	[-0.18 - 1.07]	1.57	[0.84 - 2.93]	0.19
**Turicibacter**	9	MR Egger	0.69	[-0.27 - 1.64]	1.98	[0.76 - 5.18]	0.20
		Weighted median	0.30	[-0.03 - 0.62]	1.34	[0.97 - 1.85]	0.07
		IVW	0.25	[0.01 - 0.48]	1.28	[1.01 - 1.61]	0.04
		Simple mode	0.40	[-0.07 - 0.88]	1.50	[0.93 - 2.42]	0.14
		Weighted mode	0.41	[-0.05 - 0.87]	1.50	[0.95 - 2.38]	0.12

Table S13, Supplemental Digital Content. MR estimates for the association between gut microbiota and zona pellucida sperm-binding protein 4

Bacterial genus (exposure)	No. of SNP	MR method	β	95%CI	OR	95% CI	P-value
	8	MR Egger	-0.41	[-1.42 -0.60]	0.66	[0.24 - 1.82]	0.45
		Weighted median	-0.27	[-0.58 -0.04]	0.76	[0.56 - 1.04]	0.09
**Adlercreutzia**		IVW	-0.35	[-0.60 --0.12]	0.70	[0.55 - 0.89]	0.004
		Simple mode	-0.25	[-0.70 -0.20]	0.78	[0.50 - 1.22]	0.31
		Weighted mode	-0.25	[0.72 -0.22]	0.78	[0.49 - 1.24]	0.33
	13	MR Egger	-0.71	[-1.71 - 0.30]	0.49	[0.18 - 1.35]	0.20
		Weighted median	-0.25	[-0.64 - 0.14]	0.78	[0.53 - 1.15]	0.22
**Lachnoclostridium**		IVW	-0.32	[-0.60 - -0.04]	0.73	[0.55 - 0.96]	0.03
		Simple mode	-0.18	[-0.89 - 0.53]	0.83	[0.41 - 1.70]	0.62
		Weighted mode	-0.21	[-0.93 - 0.51]	0.81	[0.40 - 1.67]	0.58
	13	MR Egger	-1.48	[-10.52 - 7.55]	0.23	[0.00 - 1896.64]	0.80
**Lachnospiraceae (ND3007 group)**		Weighted median	0.68	[-0.00 - 1.36]	1.97	[1.00 - 3.88]	0.05
		IVW	0.61	[0.06 - 1.16]	1.84	[1.06 - 3.19]	0.03
		Simple mode	0.69	[-0.08 - 1.46]	2.00	[0.93 - 4.32]	0.22
		Weighted mode	0.70	[-0.07 - 1.46]	2.01	[0.93 - 4.32]	0.22
	22	MR Egger	-0.31	[-1.51 - 0.88]	0.73	[0.22 - 2.41]	0.62
		Weighted median	0.34	[0.01 - 0.67]	1.40	[1.01 - 1.95]	0.04
**Ruminococcus (gauvreauii group）**		IVW	0.37	[0.13 - 0.62]	1.45	[1.14 - 1.85]	0.003
		Simple mode	0.31	[-0.22 - 0.84]	1.36	[0.80 - 2.31]	0.27
		Weighted mode	0.30	[-0.25 - 0.85]	1.34	[0.78 - 2.33]	0.31
	12	MR Egger	-0.26	[-0.78 - 0.26]	0.77	[0.46 - 1.29]	0.34
		Weighted median	-0.36	[-0.62 - -0.09]	0.70	[0.54 - 0.91]	0.01
**Ruminococcaceae UCG002**		IVW	-0.23	[-0.42 - -0.04]	0.80	[0.66 - 0.96]	0.02
		Simple mode	-0.44	[-0.93 - 0.06]	0.65	[0.39 - 1.06]	0.10
		Weighted mode	-0.44	[-0.98 - 0.11]	0.65	[0.37 - 1.11]	0.13
	9	MR Egger	0.93	[-0.01 - 1.87]	2.54	[0.99 - 6.51]	0.09
		Weighted median	0.15	[-0.18 - 0.47]	1.16	[0.84 - 1.61]	0.37
**Turicibacter**		IVW	0.27	[0.04 - 0.50]	1.31	[1.04 - 1.65]	0.02
		Simple mode	0.03	[-0.51 - 0.57]	1.03	[0.60 - 1.77]	0.90
		Weighted mode	0.04	[-0.49 - 0.58]	1.04	[0.61 - 1.78]	0.88

Table S14, Supplemental Digital Content. Heterogeneity in instrumental variables for gut microbiota

Disease	Bacterial genus(exposure)	Cochran's Q	df	P-value
**Inflammation**				
	Erysipelatoclostridium	14.87	14	0.39
	Faecalibacterium	12.87	9	0.17
	Lachnospiraceae UCG004	7.33	11	0.77
	Odoribacter	8.03	6	0.24
**Prostatitis**	Paraprevotella	11.92	12	0.45
	Parasutterella	7.14	13	0.89
	Ruminococcaceae UCG009	6.31	11	0.85
	Slackia	3.44	5	0.63
	Sutterella	7.60	11	0.75
**Orchitis and epididymitis**	Eubacterium (ruminantium group)	9.75	17	0.91
	Ruminococcaceae UCG009	7.87	11	0.72
**Infertility**				
	Eubacterium (oxidoreducens group)	2.30	4	0.68
**Male infertility**	Eubacterium (rectale group)	6.20	7	0.52
	Lactococcus	5.78	8	0.67
	Butyrivibrio	13.05	14	0.52
	Lachnospiraceae UCG001	6.86	11	0.81
**Abnormal spermatozoa**	Prevotella 9	18.75	14	0.17
	Ruminococcaceae UCG009	6.39	11	0.85
	Streptococcus	8.73	12	0.73
**Sperm Related Proteins**				
**SPACA3**	Defluviitaleaceae UCG011	8.67	8	0.37
**SPACA7**	Peptococcus	9.38	11	0.59
	Rikenellaceae RC9 (gut group)	10.50	10	0.40
**SPAG11A**	Anaerostipes	6.25	11	0.86
	Oxalobacter	4.90	10	0.90
	Anaerotruncus	11.26	12	0.51
	Blautia	8.77	12	0.72
**SPAG11B**	Candidatus_Soleaferrea	2.69	8	0.95
	Coprococcus 2	7.25	7	0.40
	Eubacterium (brachy group)	3.57	9	0.94
	Dorea	7.70	9	0.56
	Lactobacillus	21.13	9	0.01
**SPATA9**	Romboutsia	10.21	12	0.60
	Ruminococcaceae UCG002	17.49	21	0.68
	Ruminococcaceae UCG014	12.27	10	0.27
**SPATA20**	Subdoligranulum	4.59	10	0.92
	Turicibacter	8.13	8	0.42
	Adlercreutzia	4.41	7	0.73
	Lachnoclostridium	10.69	12	0.56
**ZPBP4**	Lachnospiraceae (ND3007 group)	0.21	2	0.90
	Ruminococcaceae UCG002	20.15	21	0.51
	Ruminococcus (gauvreauii group）	6.32	11	0.85

Turicibacter	7.66	8	0.47

Table S15, Supplemental Digital Content. Assessment of horizontal pleiotropy in the connection between gut microbiota and male reproductive health using MR Egger regression

Disease	Bacterial genus(exposure)	Egger_intercept	SE	P-value
**Inflammation**				
	Lachnospiraceae UCG004	0.04	0.05	0.44
	Odoribacter	0.01	0.07	0.90
	Paraprevotella	-0.05	0.05	0.27
	Parasutterella	0.00	0.03	0.92
**Prostatitis**	Ruminococcaceae UCG009	-0.06	0.05	0.28
	Slackia	0.15	0.11	0.24
	Sutterella	-0.06	0.05	0.26
	Erysipelatoclostridium	-0.03	0.04	0.54
	Faecalibacterium	-0.06	0.04	0.15
**Orchitis and epididymitis**	Eubacterium (ruminantium group)	-0.03	0.04	0.38
	Ruminococcaceae UCG009	-0.01	0.06	0.85
**Infertility**				
	Eubacterium (oxidoreducens group)	0.12	0.11	0.36
**Male infertility**	Eubacterium (rectale group)	0.04	0.09	0.64
	Lactococcus	-0.06	0.11	0.59
	Butyrivibrio	0.16	0.07	0.03
	Lachnospiraceae UCG001	-0.09	0.07	0.24
**Abnormal spermatozoa**	Prevotella 9	0.005	0.06	0.94
	Ruminococcaceae UCG009	-0.13	0.07	0.10
	Streptococcus	-0.06	0.07	0.38
**Sperm Related Proteins**				
**SPACA3**	Defluviitaleaceae UCG011	-0.01	0.04	0.91
**SPACA7**	Peptococcus	0.03	0.04	0.48
	Rikenellaceae RC9 (gut group)	-0.04	0.06	0.55
**SPAG11A**	Anaerostipes	0.01	0.03	0.76
	Oxalobacter	0.05	0.05	0.36
	Anaerotruncus	-0.01	0.03	0.69
	Blautia	0.02	0.02	0.34
**SPAG11B**	Candidatus_Soleaferrea	-0.01	0.10	0.96
	Coprococcus 2	-0.14	0.07	0.09
	Eubacterium (brachy group)	-0.005	0.04	0.90
	Dorea	0.01	0.03	0.83
	Lactobacillus	0.03	0.05	0.54
**SPATA9**	Romboutsia	0.01	0.03	0.84
	Ruminococcaceae UCG002	0.01	0.02	0.46
	Ruminococcaceae UCG014	0.04	0.03	0.26
**SPATA20**	Subdoligranulum	0.03	0.03	0.25
	Turicibacter	-0.05	0.05	0.39
	Adlercreutzia	0.01	0.05	0.91
**ZPBP4**	Lachnoclostridium	0.03	0.03	0.45
	Lachnospiraceae (ND3007 group)	0.12	0.27	0.73

Ruminococcaceae UCG002	0.003	0.02	0.90
Ruminococcus (gauvreauii group）	0.05	0.04	0.28
Turicibacter	-0.07	0.05	0.20

Table S16, Supplemental Digital Content. Analyzing the link between gut microbiota and male reproductive health with MR-PRESSO

Disease	Bacterial genus(exposure)	MR Analysis	Causal Estimate	SD	T-stat	P-value	RSS_{obs}	Global test P-value
**Inflammation**								
	Lachnospiraceae UCG004	MR-PRESSO	0.48	0.14	3.45	0.00	8.64	0.85
	Odoribacter	MR-PRESSO	0.31	0.25	1.20	0.26	15.91	0.17
	Paraprevotella	MR-PRESSO	0.25	0.11	2.23	0.05	14.10	0.50
	Parasutterella	MR-PRESSO	-0.29	0.11	-2.64	0.02	12.90	0.75
**Prostatitis**	Ruminococcaceae UCG009	MR-PRESSO	-0.27	0.08	-3.24	0.01	8.29	0.92
	Slackia	MR-PRESSO	-0.34	0.12	-2.73	0.03	7.45	0.69
	Sutterella	MR-PRESSO	0.46	0.14	3.26	0.01	9.37	0.75
	Erysipelatoclostridium	MR-PRESSO	-0.35	0.12	-2.80	0.01	16.82	0.48
	Faecalibacterium	MR-PRESSO	0.30	0.19	1.57	0.14	26.09	0.08
**Orchitis and epididymitis**	Eubacterium (ruminantium group)	MR-PRESSO	-0.25	0.09	-2.70	0.01	13.39	0.83
	Ruminococcaceae UCG009	MR-PRESSO	-0.31	0.12	-2.57	0.02	12.28	0.67
**Infertility**								
	Eubacterium (oxidoreducens group)	MR-PRESSO	0.47	0.30	1.55	0.18	10.68	0.20
**Male infertility**	Eubacterium (rectale group)	MR-PRESSO	-0.81	0.28	-2.85	0.02	14.36	0.40
	Lactococcus	MR-PRESSO	0.21	0.16	1.35	0.21	11.73	0.47
	Butyrivibrio	MR-PRESSO	0.18	0.10	1.80	0.09	16.36	0.52
	Lachnospiraceae UCG001	MR-PRESSO	0.27	0.17	1.56	0.14	15.02	0.53
**Abnormal spermatozoa**	Prevotella 9	MR-PRESSO	-0.40	0.17	-2.34	0.03	21.52	0.32
	Ruminococcaceae UCG009	MR-PRESSO	0.38	0.14	2.82	0.01	11.10	0.75
	Streptococcus	MR-PRESSO	0.61	0.21	2.94	0.01	11.96	0.76
**Sperm Related Proteins**								
**SPACA3**	Defluviitaleaceae UCG011	MR-PRESSO	0.19	0.11	1.76	0.11	11.99	0.38
**SPACA7**	Peptococcus	MR-PRESSO	0.15	0.07	2.16	0.05	17.28	0.45
	Rikenellaceae RC9 (gut group)	MR-PRESSO	-0.12	0.06	-1.87	0.09	15.06	0.38
**SPAG11A**	Anaerostipes	MR-PRESSO	-0.22	0.09	-2.37	0.03	8.47	0.89
	Oxalobacter	MR-PRESSO	0.14	0.05	2.90	0.01	6.42	0.93
**SPAG11B**	Anaerotruncus	MR-PRESSO	0.28	0.14	2.01	0.06	21.81	0.20

	Blautia	MR-PRESSO	0.28	0.11	2.68	0.02	9.97	0.78
	Candidatus_Soleaferrea	MR-PRESSO	-0.16	0.06	-2.61	0.02	10.21	0.84
	Coprococcus 2	MR-PRESSO	-0.24	0.14	-1.76	0.11	12.85	0.34
	Eubacterium (brachy group)	MR-PRESSO	0.18	0.04	4.04	0.00	4.39	0.98
	Dorea	MR-PRESSO	-0.34	0.13	-2.72	0.02	11.39	0.58
	Lactobacillus	MR-PRESSO	-0.21	0.13	-1.55	0.15	31.73	0.01
**SPATA9**	Romboutsia	MR-PRESSO	0.27	0.10	2.72	0.02	12.99	0.64
	Ruminococcaceae UCG002	MR-PRESSO	-0.17	0.08	-2.08	0.05	21.08	0.77
	Ruminococcaceae UCG014	MR-PRESSO	0.25	0.11	2.32	0.03	21.60	0.34
**SPATA20**	Subdoligranulum	MR-PRESSO	0.16	0.15	1.06	0.31	20.64	0.17
	Turicibacter	MR-PRESSO	0.23	0.10	2.36	0.03	15.57	0.45
	Adlercreutzia	MR-PRESSO	-0.31	0.08	-3.82	0.00	8.90	0.76
	Lachnoclostridium	MR-PRESSO	-0.19	0.14	-1.30	0.22	19.30	0.28
**ZPBP4**	Lachnospiraceae (ND3007 group)	MR-PRESSO	0.44	0.18	2.45	0.09	3.02	0.67
	Ruminococcaceae UCG002	MR-PRESSO	-0.23	0.09	-2.68	0.01	25.27	0.56
	Ruminococcus (gauvreauii group）	MR-PRESSO	0.37	0.09	3.93	0.00	7.49	0.87
	Turicibacter	MR-PRESSO	0.29	0.07	3.88	0.00	9.09	0.87
