# Supplementary material for: Causal relationships between gut microbiota and male reproductive inflammation and infertility: Insights from Mendelian randomization
Source: Medicine (Baltimore). 2025 Apr 25;104(17):e42323. doi: 10.1097/MD.0000000000042323 (PMC12039986; doi:10.1097/MD.0000000000042323)
Supplement: Supplementary file 2 [file medi-104-e42323-s002.pdf]

Figure S1, Supplemental Digital Content

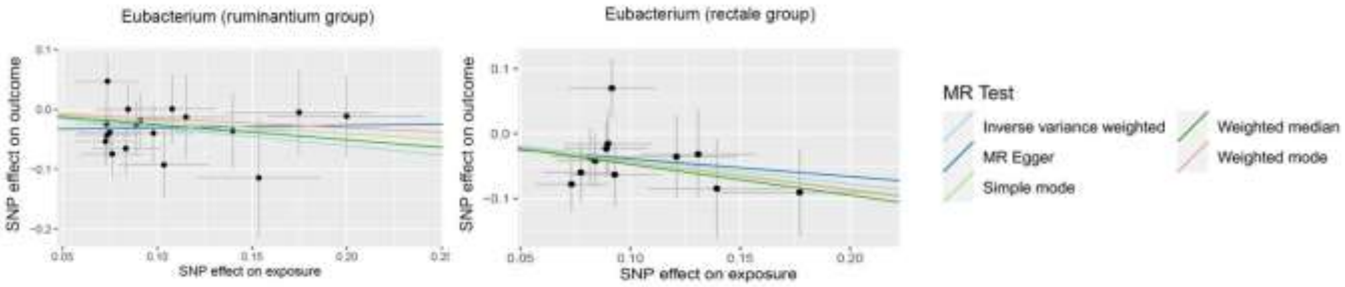

Figure S1, Supplemental Digital Content Scatter plot of the causal association between gut microbiome and orchitis and epididymitis. SNPs = single nucleotide polymorphisms.

Figure S2, Supplemental Digital Content

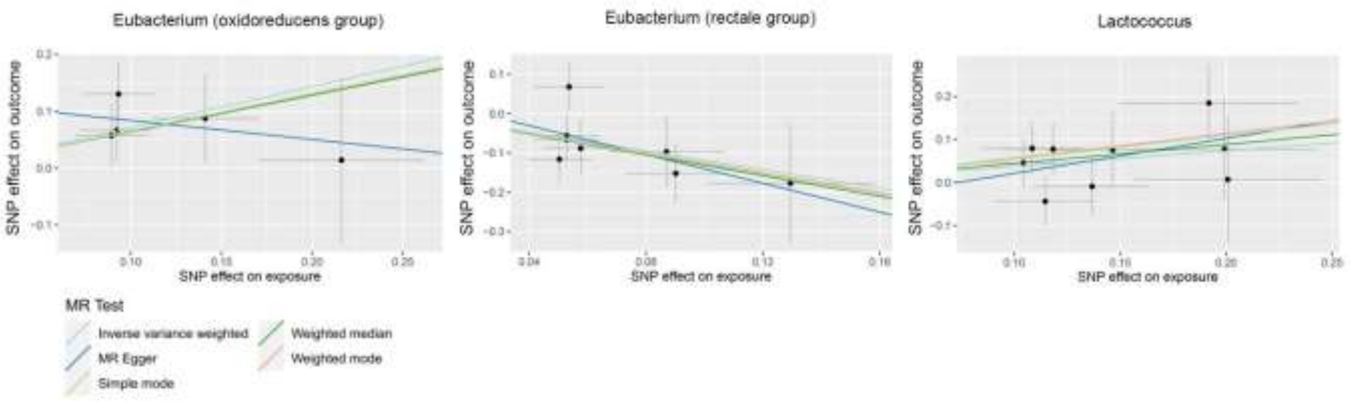

Figure S2, Supplemental Digital Content Scatter plot of the causal association between gut microbiome and male infertility. SNPs = single nucleotide polymorphisms.

Figure S3, Supplemental Digital Content

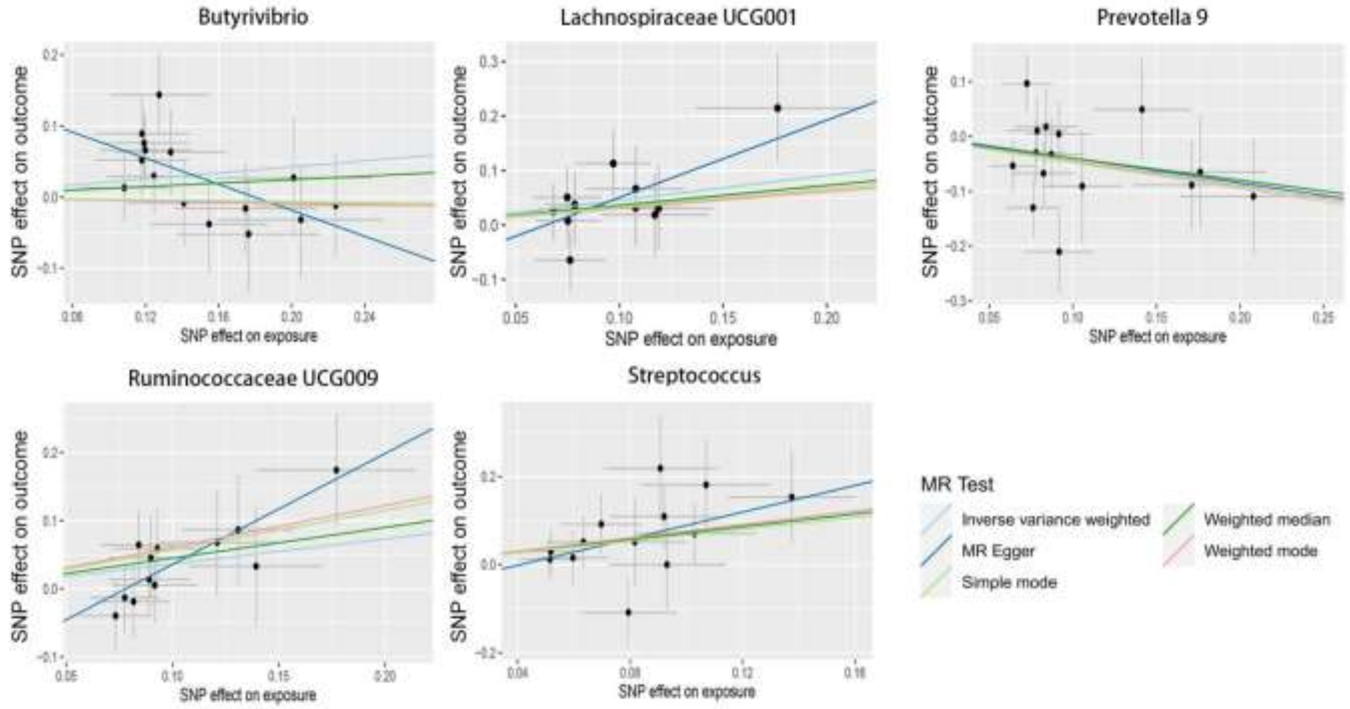

Figure S3, Supplemental Digital Content Scatter plot of the causal association between gut microbiome and abnormal spermatozoa. SNPs = single nucleotide polymorphisms.

Figure S4, Supplemental Digital Content

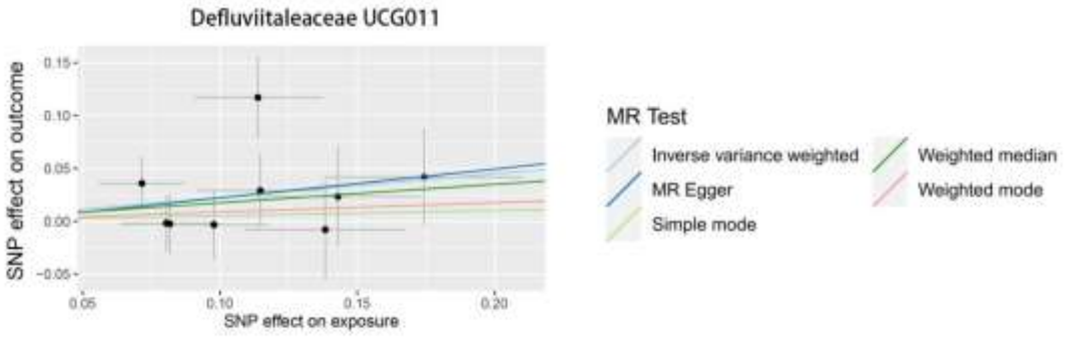

Figure S4, Supplemental Digital Content Scatter plot of the causal association between gut microbiome and SPACA3. SNPs = single nucleotide polymorphisms; SPACA3 = sperm acrosome membrane-associated protein 3.

Figure S5, Supplemental Digital Content

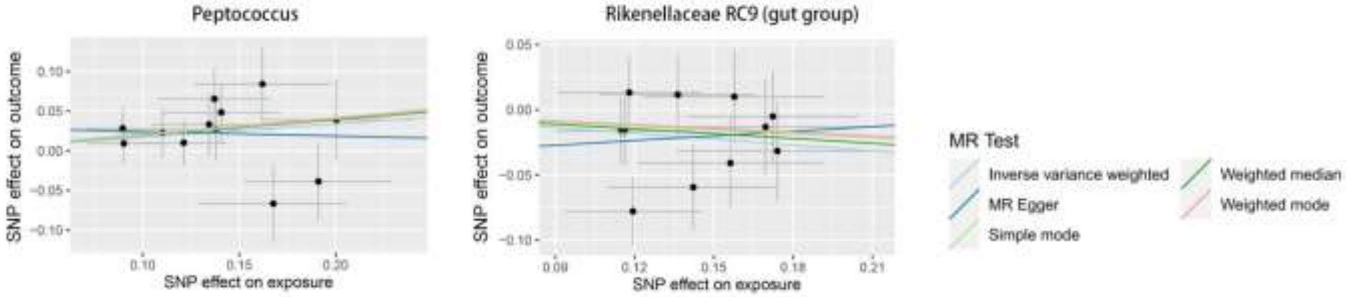

Figure S5, Supplemental Digital Content Scatter plot of the causal association between gut microbiome and SPACA7. SNPs = single nucleotide polymorphisms; SPACA7 = sperm acrosome associated 7.

Figure S6, Supplemental Digital Content

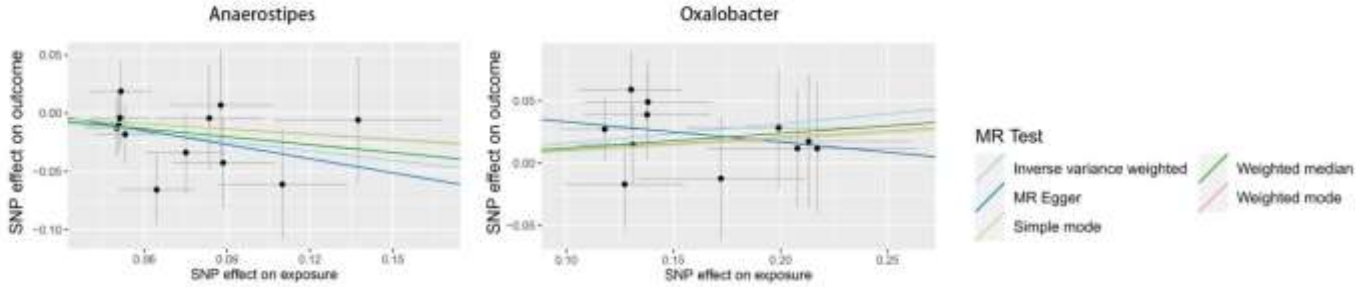

Figure S6, Supplemental Digital Content Scatter plot of the causal association between gut microbiome and SPAG11A. SNPs = single nucleotide polymorphisms; SPAG11A = sperm-associated antigen 11A.

Figure S7, Supplemental Digital Content

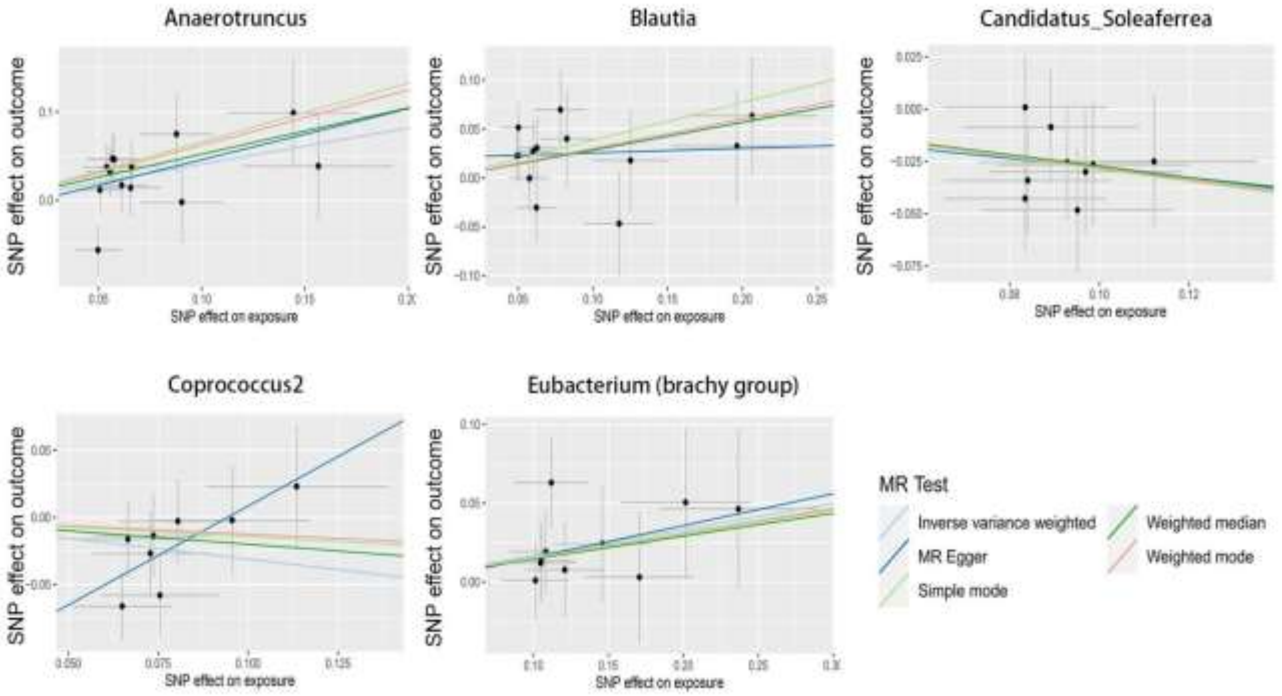

Figure S7, Supplemental Digital Content Scatter plot of the causal association between gut microbiome and SPAG11B. SNPs = single nucleotide polymorphisms; SPAG11B = sperm-associated antigen 11B.

Figure S8, Supplemental Digital Content

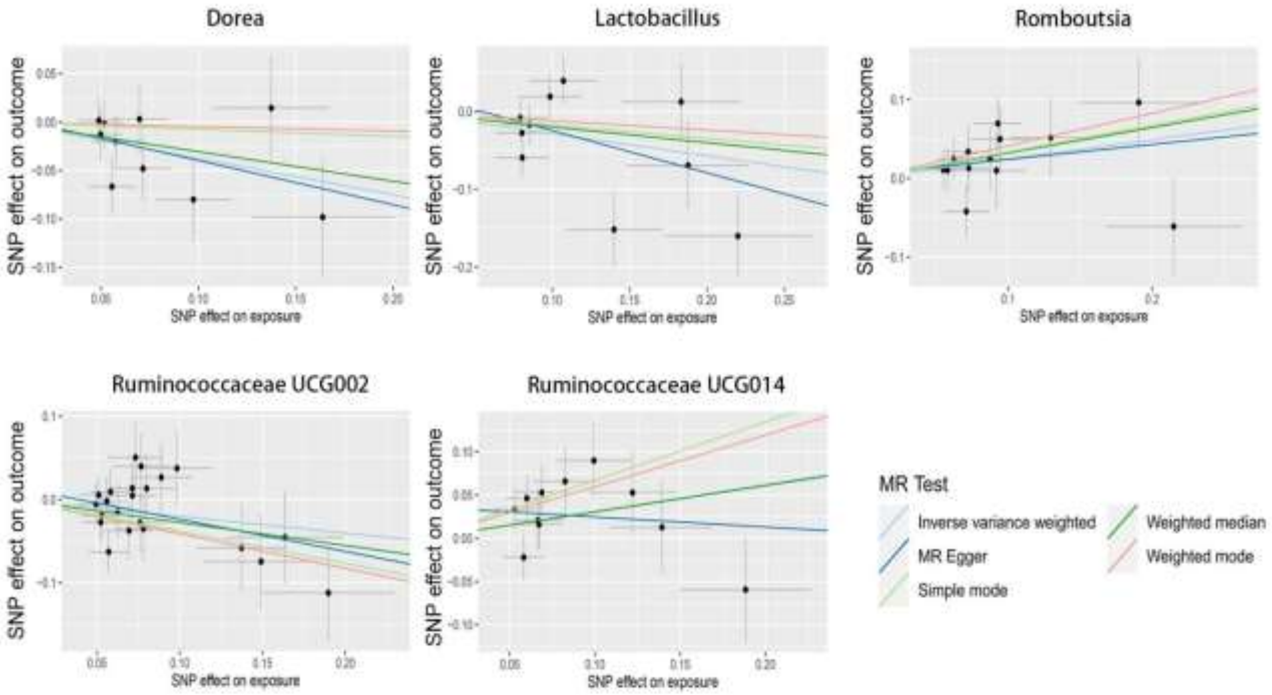

Figure S8, Supplemental Digital Content Scatter plot of the causal association between gut microbiome and SPATA9. SNPs = single nucleotide polymorphisms, SPATA9 = spermatogenesis-associated protein 9.

Figure S9, Supplemental Digital Content

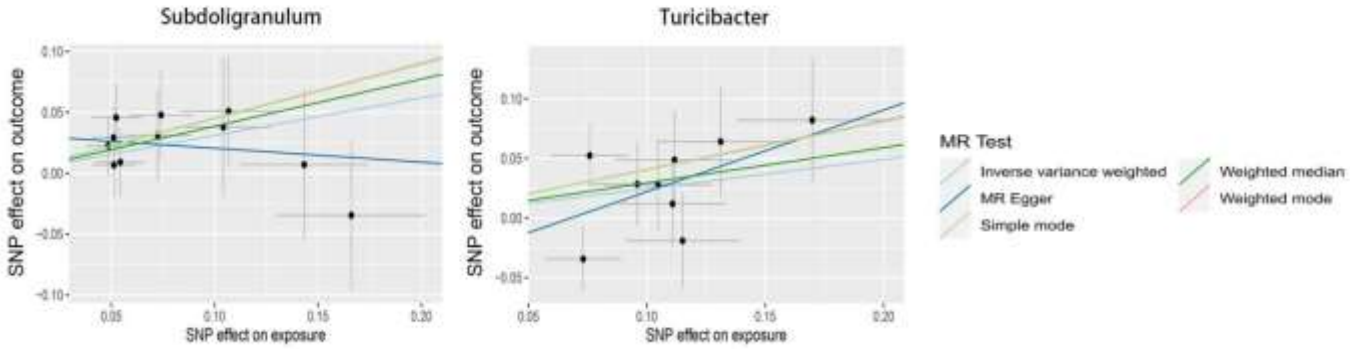

Figure S9, Supplemental Digital Content Scatter plot of the causal association between gut microbiome and SPATA20. SNPs = single nucleotide polymorphisms; SPATA20 = spermatogenesis-associated protein 20.

Figure S10, Supplemental Digital Content

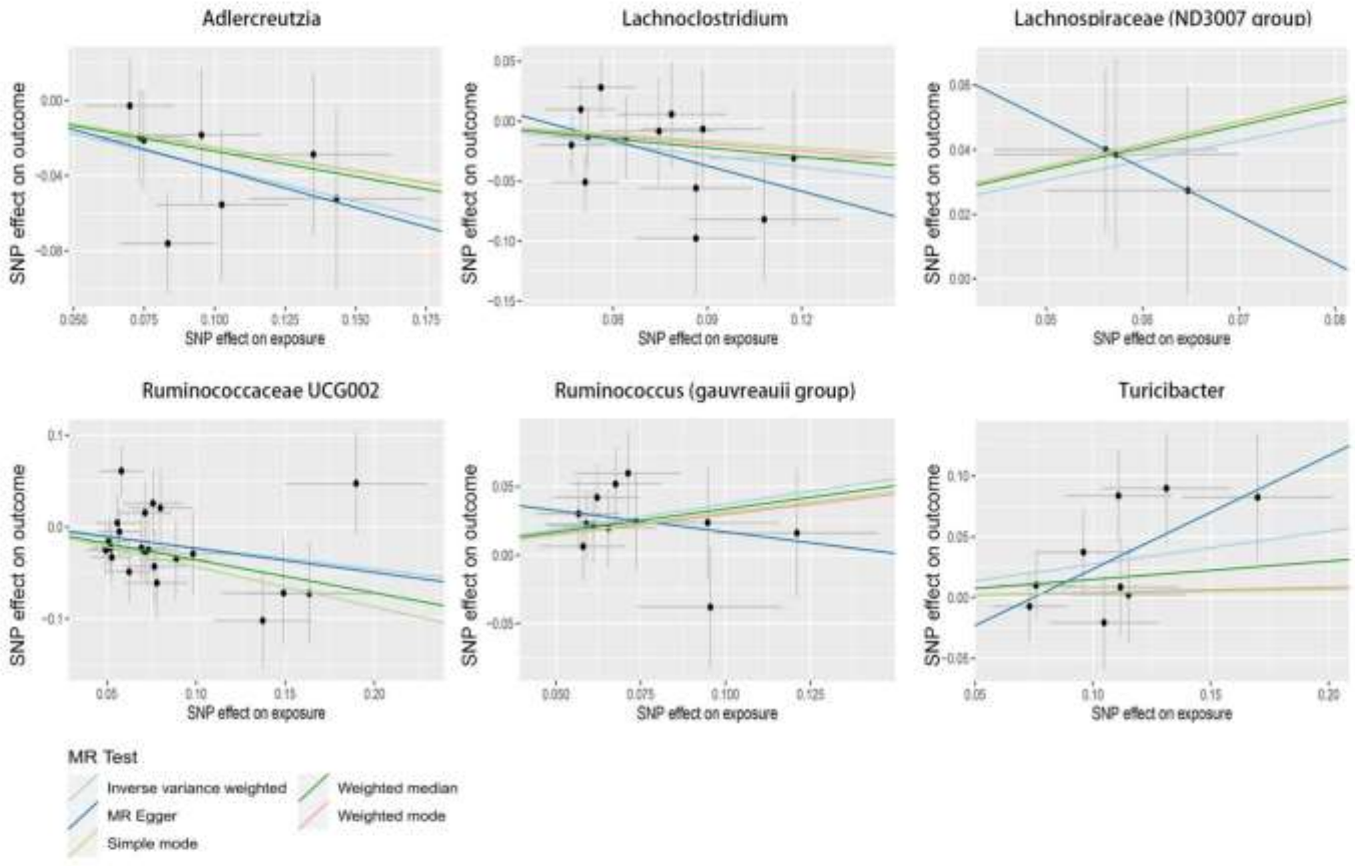

Figure S10, Supplemental Digital Content Scatter plot of the causal association between gut microbiome and ZPBP4. SNPs = single nucleotide polymorphisms; ZPBP4 = zona pellucida sperm-binding protein 4.

Figure S11, Supplemental Digital Content

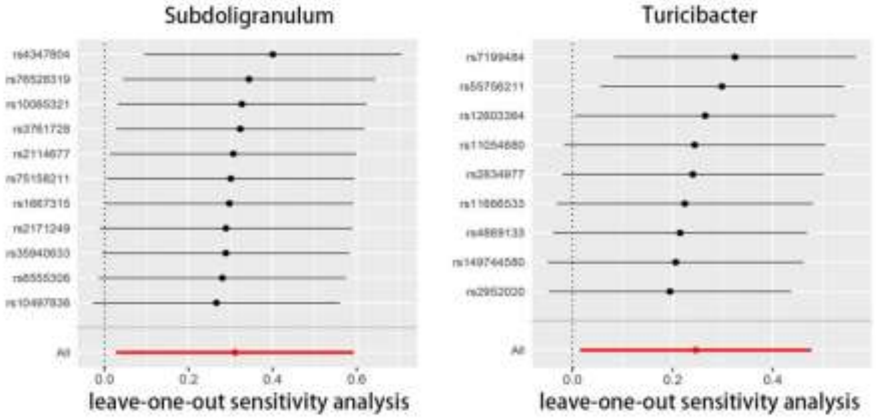

Figure S11, Supplemental Digital Content Leave-one-out analysis of the causal association between gut microbiome and orchitis and epididymitis.

Figure S12, Supplemental Digital Content

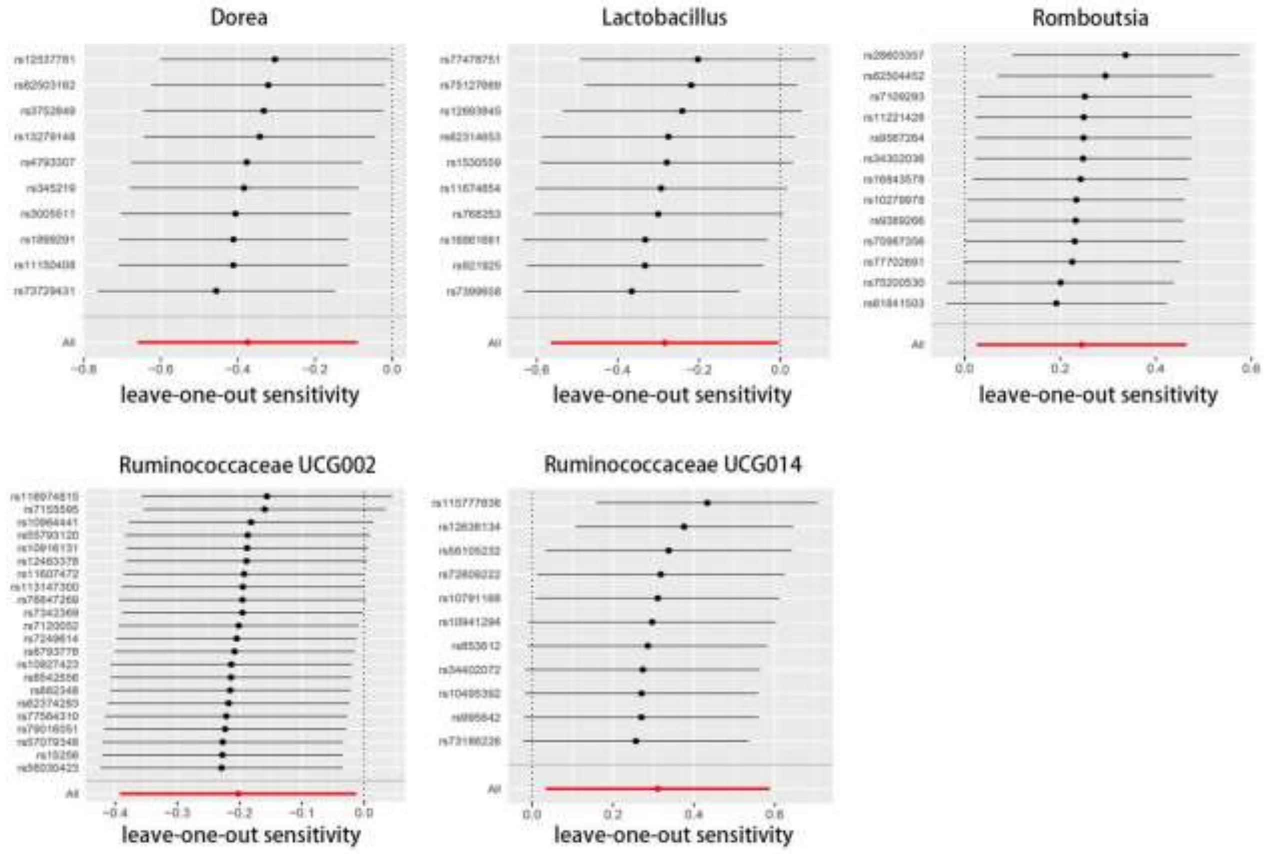

Figure S12, Supplemental Digital Content Leave-one-out analysis of the causal association between gut microbiome and male infertility.

Figure S13, Supplemental Digital Content

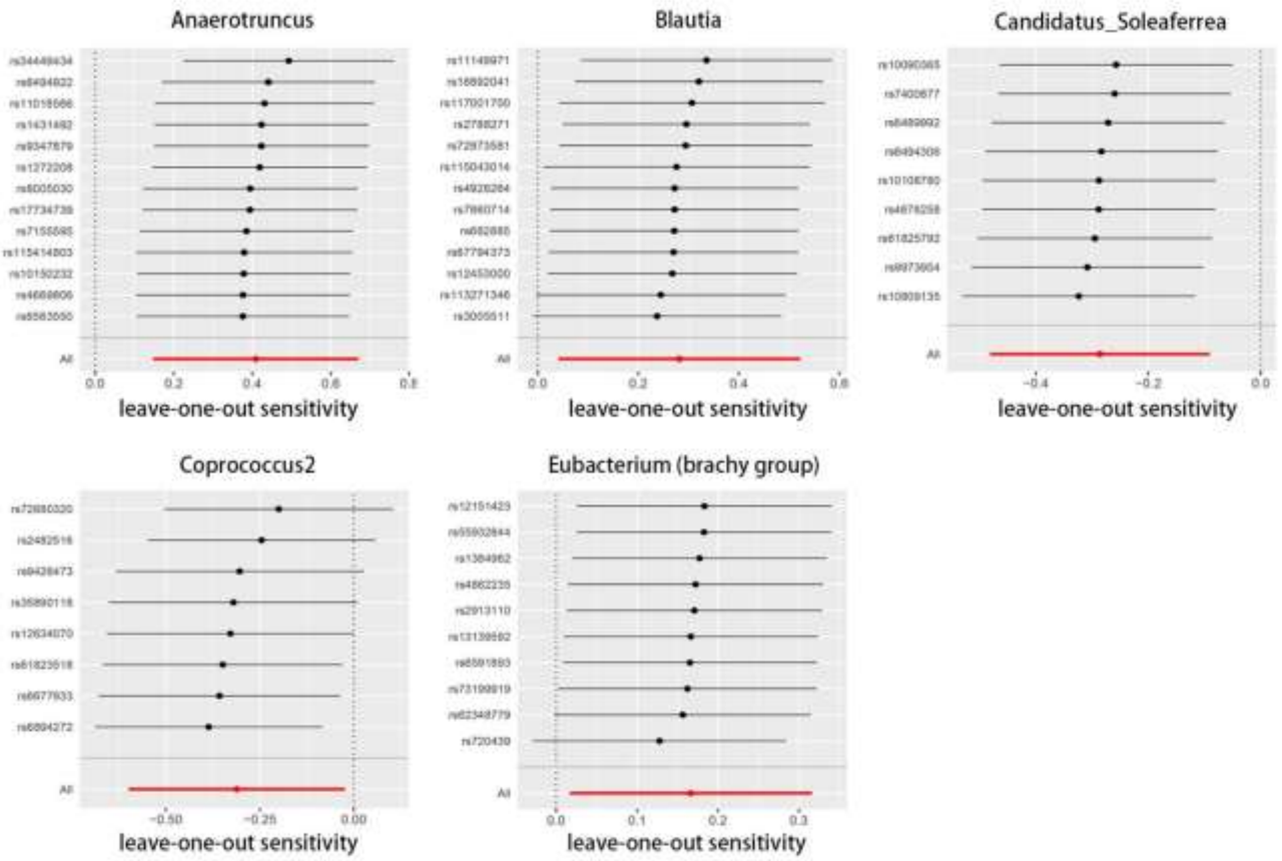

Figure S13, Supplemental Digital Content Leave-one-out analysis of the causal association between gut microbiome and abnormal spermatozoa.

Figure S14, Supplemental Digital Content

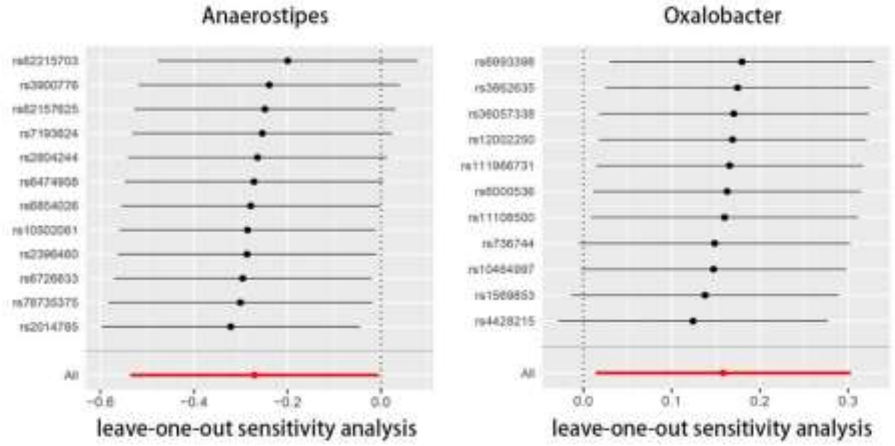

Figure S14, Supplemental Digital Content Leave-one-out analysis of the causal association between gut microbiome and SPACA3. SPACA3 = sperm acrosome membrane-associated protein 3.

Figure S15, Supplemental Digital Content

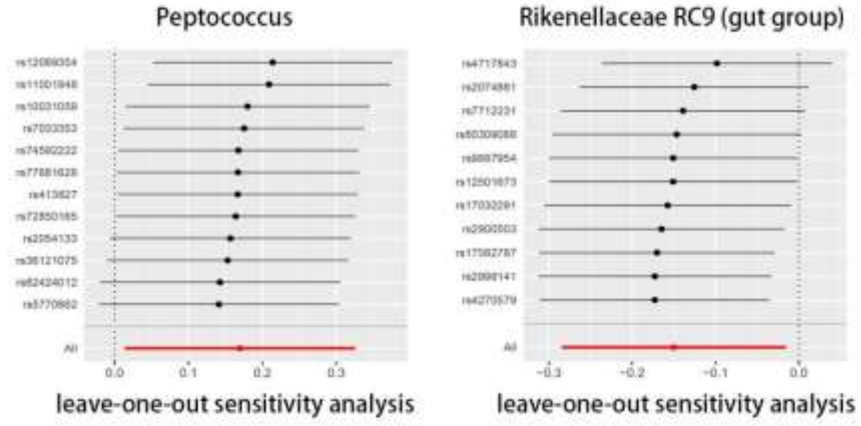

Figure S15, Supplemental Digital Content Leave-one-out analysis of the causal association between gut microbiome and SPACA7. SPACA7 = sperm acrosome associated 7.

Figure S16, Supplemental Digital Content

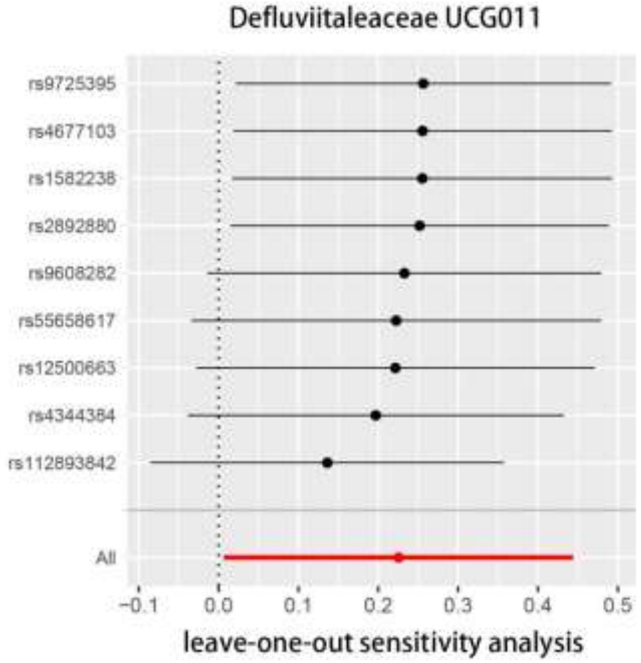

Figure S16, Supplemental Digital Content Leave-one-out analysis of the causal association between gut microbiome and SPAG11A. SPAG11A = sperm-associated antigen 11A.

Figure S17, Supplemental Digital Content

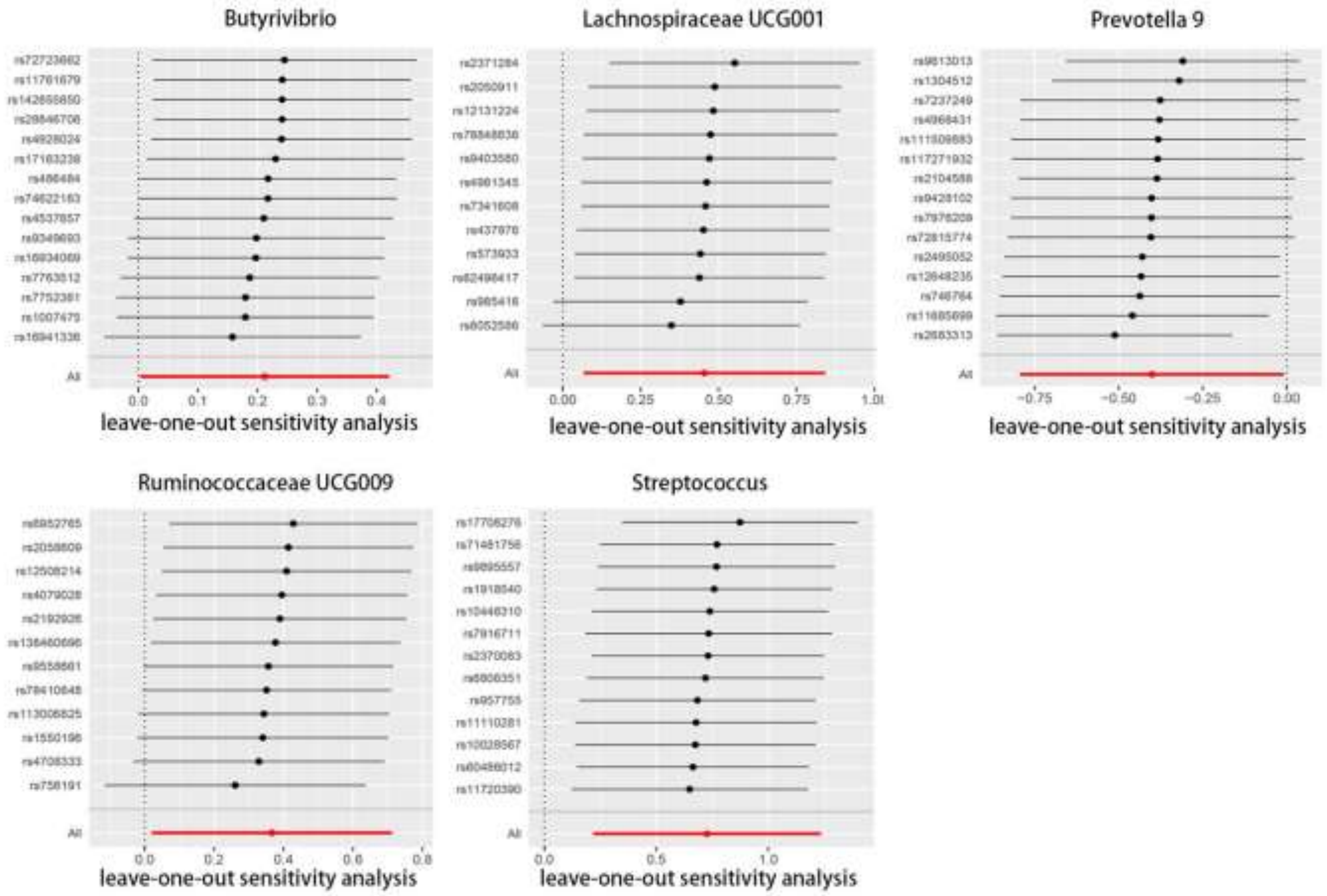

Figure S17, Supplemental Digital Content Leave-one-out analysis of the causal association between gut microbiome and SPAG11B. SPAG11B = sperm-associated antigen 11B.

Figure S18, Supplemental Digital Content

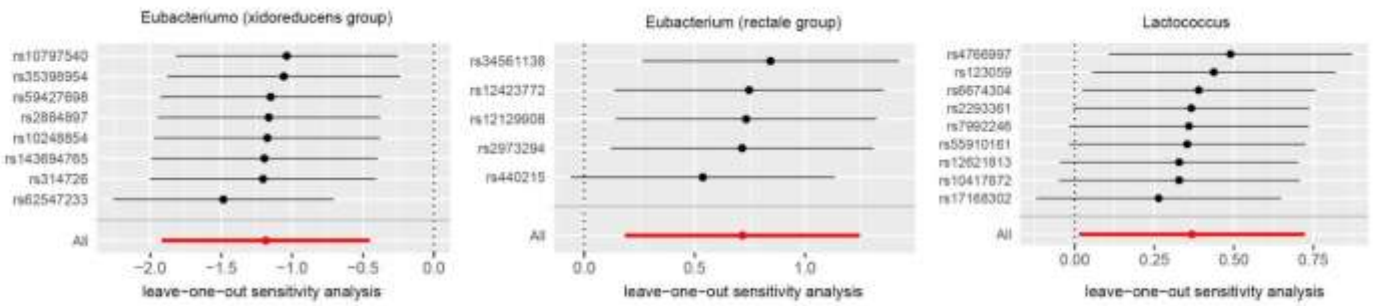

Figure S18, Supplemental Digital Content Leave-one-out analysis of the causal association between gut microbiome and SPATA9. SPATA9 = spermatogenesis-associated protein 9.

Figure S19, Supplemental Digital Content

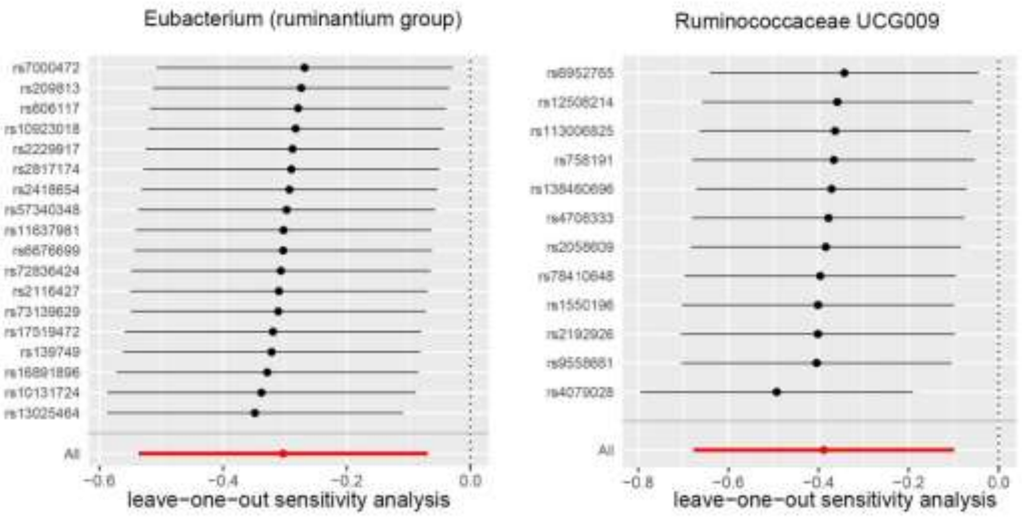

Figure S19, Supplemental Digital Content Leave-one-out analysis of the causal association between gut microbiome and SPATA20. SPATA20 = spermatogenesis-associated protein 20.

Figure S20, Supplemental Digital Content

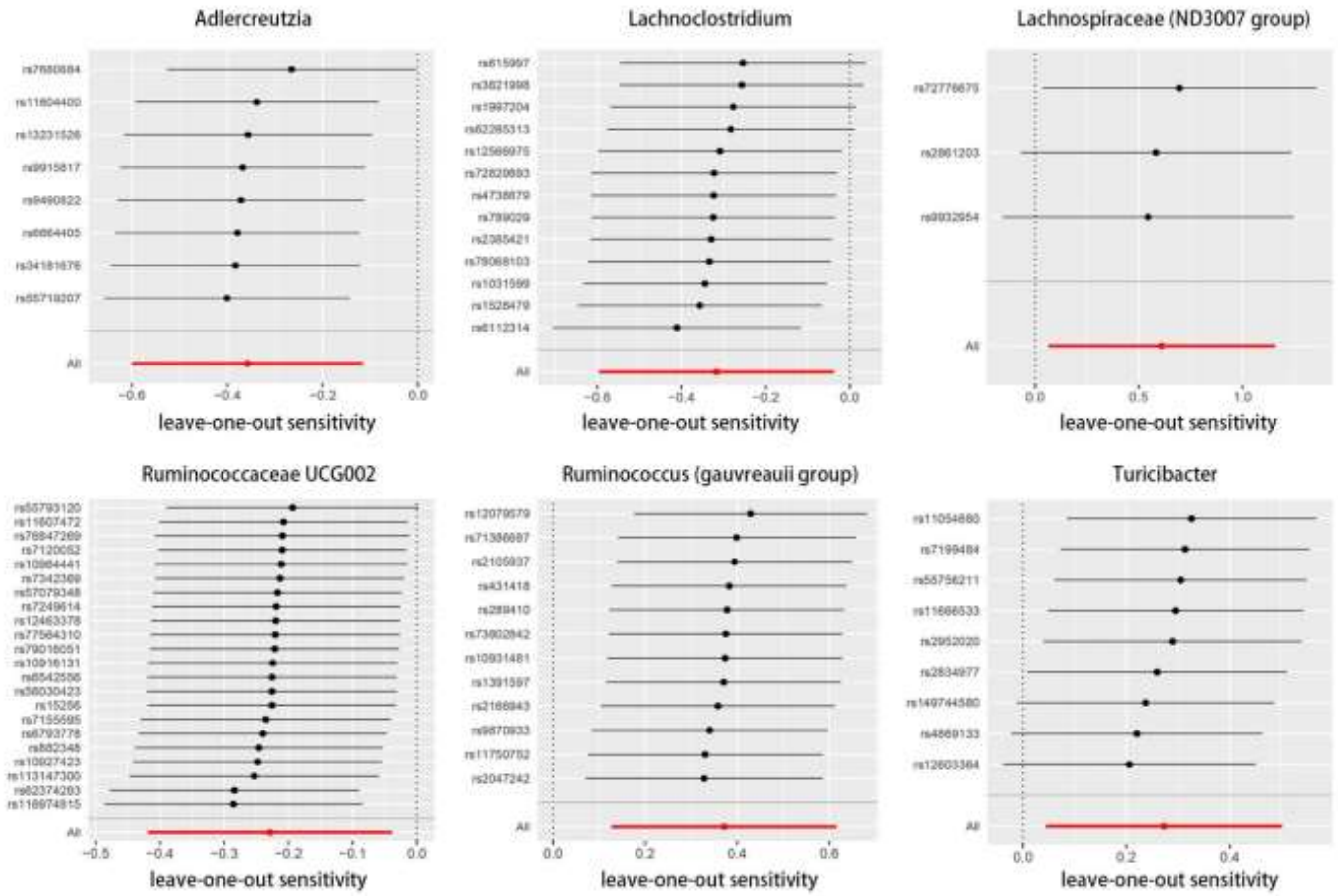

Figure S20, Supplemental Digital Content Leave-one-out analysis of the causal association between gut microbiome and ZPBP4. ZPBP4 = zona pellucida sperm-binding protein 4.
